# A Multifunctional Integrated Metal‐Free MRI Agent for Early Diagnosis of Oxidative Stress in a Mouse Model of Diabetic Cardiomyopathy

**DOI:** 10.1002/advs.202206171

**Published:** 2023-01-03

**Authors:** Zhuang Nie, Kun Zhang, Xinyu Chen, Jingxin Wang, Huile Gao, Bingwen Zheng, Qihong Wu, Yingkun Guo, Xiangyang Liu, Xu Wang

**Affiliations:** ^1^ College of Polymer Science and Engineering State Key Laboratory of Polymer Material and Engineering Sichuan University Chengdu 610065 P. R. China; ^2^ Department of Radiology Key Laboratory of Birth Defects and Related Diseases of Women and Children of Ministry of Education West China Second University Hospital Sichuan University 20# South Renmin Road Chengdu Sichuan 610041 P. R. China; ^3^ Key Laboratory of Drug‐Targeting and Drug Delivery System of the Education Ministry Sichuan Engineering Laboratory for Plant‐Sourced Drug and Sichuan Research Center for Drug Precision Industrial Technology West China School of Pharmacy Sichuan University Chengdu 610064 P. R. China; ^4^ Time Medical Ltd., Hong Kong Science & Technology Park Hong Kong 999077 P. R. China

**Keywords:** diabetic cardiomyopathy, fluorinated carbon materials, magnetic resonance imaging, multifunctional integrated probe, reactive oxygen species

## Abstract

Reactive oxygen species (ROS) are closely associated with the progression of diabetic cardiomyopathy (DCM) and can be regarded as one of its early biomarkers. Magnetic resonance imaging (MRI) is emerging as a powerful tool for the detection of cardiac abnormalities, but the sensitive and direct ROS‐response MRI probe remains to be developed. This restricts the early diagnosis of DCM and prevents timely clinical interventions, resulting in serious and irreversible pathophysiological abnormalities. Herein, a novel ROS‐response contrast‐enhanced MRI nanoprobe (RCMN) is developed by multi‐functionalizing fluorinated carbon nanosheets (FCNs) with multi‐hydroxyl and 2,2,6,6‐tetramethylpiperidin‐1‐oxyl groups. RCMNs capture ROS and then gather in the heart provisionally, which triggers MRI signal changes to realize the in vivo detection of ROS. In contrast to the clinical MRI agents, the cardiac abnormalities of disease mice is detected 8 weeks in advance with the assistance of RCMNs, which greatly advances the diagnostic window of DCM. To the best of the knowledge, this is the first ROS‐response metal‐free T_2_‐weighted MRI probe for the early diagnosis of DCM mice model. Furthermore, RCMNs can timely scavenge excessively produced ROS to alleviate oxidative stress.

## Graphical abstract

This is a novel, metal‐free ROS‐sensitive MRI probe (RCMNs) for the early diagnosis of DCM in mice models by using the FCNs as functional carriers. Compared with conventional MRI agents, the cardiac abnormalities are detected 8 weeks in advance with assistance of RCMNs. Furthermore, RCMNs can timely scavenge excessively produced ROS to alleviate oxidative stress.

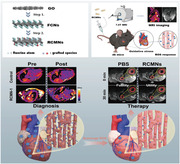

## 1 Introduction

Diabetes, one of the leading causes of death worldwide, seriously threatens human health by inducing cardiovascular complications, such as diabetic cardiomyopathy (DCM), coronary artery disease, and heart failure.^[^ ^1^, ^2^, ^3^ ^]^ Among them, DCM has been implicated as one of the most important contributors to heart failure and death in diabetic patients.^[^ ^4^, ^5^, ^6^ ^]^ Nevertheless, current clinical diagnoses of DCM are available only at its moderate and advanced stages when the body has been unfortunately undergoing certain irreversible pathophysiological abnormalities, like myocardial fibrosis.^[^ ^7^, ^8^ ^]^ Because of the lag time between the onset of DCM and current diagnoses, it is difficult to implement timely clinical interventions to reverse the pathological progress. Therefore, it appears to be particularly urgent to develop feasible approaches that enable sensitive detection of early pathological evolution of DCM.

Long‐term oxidative stress in patients with diabetes is one of important factors leading to myocardial dysfunction and closely associated with the occurrence and progression of DCM.^[^ ^9^, ^10^, ^11^ ^]^ Excessive production and accumulation of ROS could result in further membrane and DNA damage, protein degeneration, cardiomyocyte apoptosis, microvascular damage, chronic inflammation, and myocardial fibrosis.^[^ ^9^, ^12^, ^13^ ^]^ Hence, ROS may serve as a molecular biomarker in the early stage of DCM for a systematic diagnosis and therapy.^[^ ^14^ ^]^ However, the detection of ROS distribution in situ is difficult because of its short half‐life (nanoseconds to milliseconds).^[^ ^15^, ^16^ ^]^ Recently, several imaging modalities have been used for the in vivo imaging of ROS, while there are still some disadvantages. For example, optical imaging is limited in its penetration depth,^[^ ^17^, ^18^ ^]^ and ultrasound as well as photoacoustic imaging cannot provide precise tissue anatomy information.^[^ ^19^, ^20^ ^]^ Nuclear imaging requires the use of radioactivity and has a poor resolution.^[^ ^21^ ^]^ By comparison, MRI is extensively applied to detect cardiac structural abnormalities, owing to its non‐ionizing radiation and imaging at high spatial resolution.^[^ ^22^, ^23^ ^]^ MRI often requires exogenous contrast agents (CAs) to improve diagnostic accuracy. Currently, widely used MRI CAs are divided into T_1_ and T_2_ CAs. Among them, T_1_ CAs are mainly gadolinium‐based chelates, and the main component of T_2_ CAs is superparamagnetic iron oxide nanoparticles (SPIONs), which shorten the longitudinal and transverse relaxation times of water protons, respectively.^[^ ^24^, ^25^ ^]^ Unfortunately, commercially available CAs are unable to monitor ROS at the molecular level and thus can only show positive results in the late stage of DCM with perfusion defects. Besides, both T_1_ and T_2_ CAs contain heavy‐metal elements, which can induce potential safety risks, such as renal toxicity (Gd‐based CAs) and allergic reactions (SPIONs).^[^ ^25^, ^26^, ^27^, ^28^, ^29^ ^]^ Therefore, developing safe and non‐toxic probes with high ROS sensitivity and MRI contrast enhancement is highly desirable for the early diagnosis of DCM.^[^ ^29^, ^30^, ^31^ ^]^

**Scheme 1:**
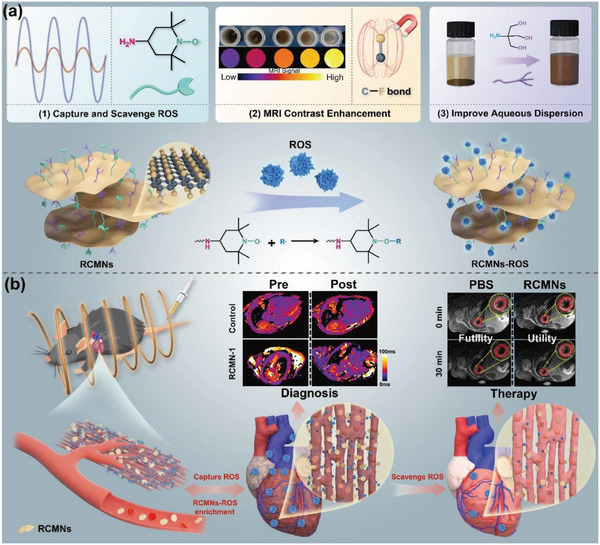
Schematic illustration of ROS‐response contrast‐enhanced MRI nanoprobes for the early diagnosis and further therapy of diabetic cardiomyopathy. a) The introduction of multiple hydroxyl groups onto their surface greatly improved the aqueous dispersion of functionalized FCNs, and the coexistence of TEMPO moieties and C—F bonds successfully combined the ROS‐sensitive property and the high resolution and the anatomical information deriving from MRI. b) When exposed to ROS‐rich microenvironment of the injured hearts, RCMNs nanoprobes were actually functionalized by the captured ROS groups and equipped with a stronger interaction with the surrounding microenvironment. This promoted a brief enrichment of the material in the myocardium, caused a variation of the corresponding MRI signal and thus realized the in vivo MRI imaging of ROS. Furthermore, the TEMPO moieties of RCMNs could scavenge ROS to alleviate oxidative stress.

Herein, we developed a novel metal‐free ROS‐response contrast‐enhanced MRI nanoprobe (RCMN) for early diagnosis of DCM based on the multifunctional integration of fluorinated carbon nanosheets (FCNs). As the amino‐terminal nucleophiles attacked C—F bonds, ROS‐response TEMPO species and multiple hydroxyl groups were integrated onto FCNs (**Scheme** **1**).^[^ ^32^, ^33^, ^34^, ^35^ ^]^ The residual C–F bonds provided the nanoprobe with MRI contrast enhancement.^[^ ^36^, ^37^ ^]^ The introduction of multiple hydroxyl groups greatly improved the water dispersibility of FCNs. Moreover, the coexistence of TEMPO moieties and C—F bonds successfully combined the ROS‐sensitive property and the high‐resolution anatomical information deriving from MRI. When exposed to ROS‐rich microenvironment of the injured hearts, ROS‐response TEMPO moieties of RCMNs captured excessive ROS. This promoted the enrichment of the probes in the heart provisionally and thus caused a variation of the corresponding MRI signal.^[^ ^38^, ^39^, ^40^ ^]^ Using conventional gadolinium contrast agents, MRI‐positive perfusion defects could be identified at the 16th week of DCM mice models.^[^ ^41^ ^]^ By contrast, the RCMN probe allowed diagnosis at the 8th week, which provides more time for follow‐up treatment. Furthermore, TEMPO moieties of RCMN could effectively remove part of the excessively produced ROS,^[^ ^40^ ^]^ and alleviate the damage caused by it. The oxidative stress indicators of the treated mice were significantly lower than those of the control mice. To the best of our knowledge, this is the first ROS‐response carbon‐based MRI probe material that has been demonstrated for the early diagnosis and timely treatment of DCM in model mice.

## 2 Results and Discussion

### 2.1 Preparation and Characterization of RCMNs

To meet the aforementioned demands for the early diagnosis of DCM, the MRI agent materials are required to exhibit the characteristics of ROS sensitivity, MRI contrast enhancement, dispersion stability and favorable biocompatibility, simultaneously. It is obviously challenging to design and obtain the multifunction integrated materials.

**Figure 1:**
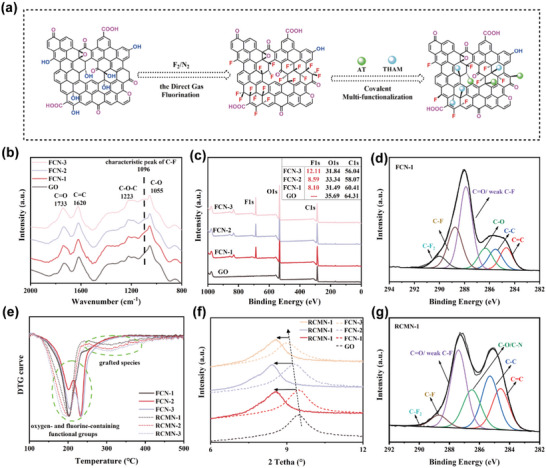
Structure characterization and schematic of the formation process for different FCNs and RCMNs. a) Schematic of the formation process for RCMN. Firstly, FCNs were fabricated with graphene oxide (GO) as raw materials via direct gas fluorination. Next, utilizing the chemical reactivity of the introduced C—F bonds, AT and THAM were covalently grafted onto FCNs’ carriers, and eventually RCMN nanoprobes were obtained. FTIR spectra b) and XPS survey spectra c) of GO and different FCNs. d) XPS C1s spectra of FCN‐1. e) DTG curves of FCNs and RCMNs samples at a heating rate of 10 °C min^−1^ under N_2_ atmosphere. f) XRD patterns of GO, different FCNs and corresponding RCMNs. g) XPS C1s spectra of RCMN‐1.

Due to prominent physicochemical properties,^[^ ^29^, ^30^, ^42^ ^]^ fluorinated carbon nanosheets (FCNs) and their derivatives have received intensive attention in biomedical fields, especially their potential application for multimodal imaging.^[^ ^36^, ^43^ ^]^ Apart from their intrinsic characteristics, fluorinated carbon materials can also go through flexible derivative reactions for further chemical modification to satisfy the needs of functional and even multifunctional applications.^[^ ^44^, ^45^, ^46^, ^47^ ^]^ Herein, FCNs were initially fabricated using graphene oxide (GO) as raw materials via direct gas fluorination (**Figure** **1**a),^[^ ^48^, ^49^ ^]^ which can facilely convert the original carbon nanosheets into FCNs with a diverse fluorination degree. Considering the effects of fluorine and corresponding C—F bonds on the paramagnetic behavior and hydrophobicity, we prepared three types of FCNs (FCN‐1, FCN‐2, and FCN‐3) with low, medium, and high degrees of fluorination, respectively (the detailed fabrication process was described in the Supporting Information). To confirm whether the F atoms were introduced, we first applied Fourier transform infrared (FTIR) spectroscopy to analyze the microstructural evolvement of GO nanosheets before and after fluorination (Figure 1b). Strong peaks were observed at 1733, 1620, 1223, and 1055 cm^−1^ in GO and FCNs, which are assigned to the vibrations of their C=O, C=C, C—O—C, and C—O bonds. Remarkably, a new peak emerged at 1096 cm^−1^, characteristic absorption of the C—F bonds.^[^ ^50^ ^]^ Hence, the successful introduction of F atoms was demonstrated. In the meantime, the intensity of the C=C stretching peak got weaker with ongoing fluorination. The presence of F atoms was further supported via X‐ray photoelectron spectroscopy (XPS) spectra (Figure 1c,d; Figure S1a–c, Supporting Information) and X‐ray diffraction (XRD) patterns (Figure 1f). XPS survey spectra revealed intensive F1s peaks in FCN‐1, FCN‐2, and FCN‐3. After deconvolution, their F/C ratios were 0.134, 0.148, and 0.216, respectively (Figure 1c). Additionally, different fluorocarbon species coexisted in FCNs (C—F, C—F2, and weak C—F),^[^ ^51^, ^52^ ^]^ as shown in the C1s (Figure 1d; Figure S1b,c, Supporting Information) and F1s (Figure S6a–c, Supporting Information) spectra of XPS. Moreover, XRD patterns suggested an obvious difference between pristine GO and FCNs with different F/C ratios (Figure 1f). The diffraction peak of pristine GO (2*θ* = 9.777°, d‐spacing = 0.904 nm) shifted progressively toward a lower angle as the fluorination proceeded, indicating that the interlayer distance was augmented by generated C—F bonds, and d‐spacing could be as high as 0.981 nm (FCN‐3).

Successfully prepared FCNs laid the foundation for designing and fabricating RCMNs. On the one hand, the introduced C—F bonds are supposed to endow FCNs with localized paramagnetic centers, making it possible to confer MRI contrast.^[^ ^36^, ^37^ ^]^ On the other hand, C—F bonds have been confirmed to chemically react with nucleophilic reagents.^[^ ^29^, ^35^ ^]^ Thus, FCNs can undergo various grafting reactions for further functional and even multifunctional decoration on the surface. Nevertheless, successful grafting reactions are always accompanied by severe reductive defluorination,^[^ ^30^, ^53^ ^]^ resulting in the recovery of the graphene structure (Figure S2, Supporting Information). As shown in Movie S1 (Supporting Information), under the attack of nucleophilic reagent, a part of C‐F bonds in FCNs are broken, the color of the reaction mixture rapidly became dark. To optimize the reaction conditions, we evaluated the reaction of FCNs with amino‐terminal nucleophiles at different ratios, graft species, and temperatures (Figure S3 and Table S2, Supporting Information). The reaction kinetics experiments demonstrated that under the premise of ensuring successful grafting, a lower temperature could suppress reductive defluorination to some extent (Figure S3, Supporting Information). Additionally, the contribution of tris(hydroxymethyl)aminomethane (THAM) to improve the aqueous dispersity was confirmed by comparing with the materials unequipped with THAM, as shown in Table S2 and Figure S4 (Supporting Information). It was found that the introduction of multiple hydroxyl groups significantly improved FCNs’ dispersion in the aqueous medium, conducive to the subsequent tests. Thus, to obtain the materials with ROS sensitivity, good hydrophilicity and relatively higher fluorine content, RCMNs were prepared by reacting FCNs with 4‐amino‐TEMPO (AT) and THAM at a temperature of ≈−45 °C (see details in the Supporting Information).

**Figure 2:**
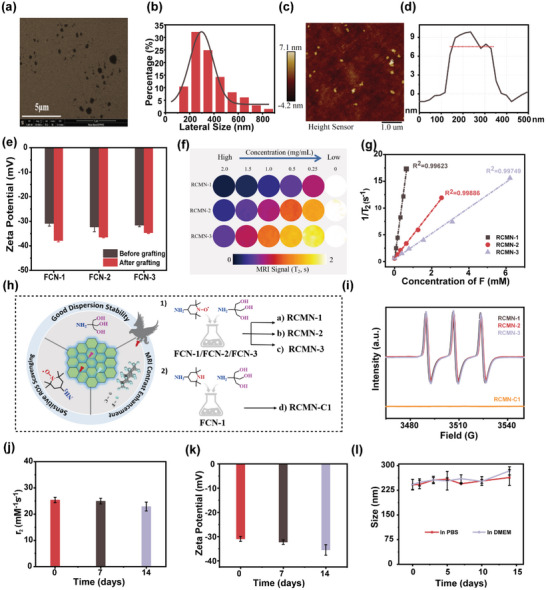
a) Schematic illustration of the functions of different groups as well as chemical structure of corresponding nanoprobes. SEM image a), statistical analysis of the particle size distribution b), scale bar: 5 µm; and AFM image c) and the corresponding height profiles of RCMNs d), scale bar: 1 µm. e) Changes in zeta potentials of FCNs before and after grafting. f) T_2_‐weighted images of RCMN‐1, RCMN‐2 and RCMN‐3 in phosphate buffered saline (PBS) medium with the concentration of RCMNs decreasing gradually from 2 mg mL^−1^ to 0, and corresponding T_2_ relaxation rates (1/T_2_) plotted against F concentrations g). h) Schematic diagram for the multifunctional integration strategy and the fabrication of different nanoprobes. i) Electron paramagnetic resonance (EPR) spectra of different RCMNs and the control sample RCMN‐C1. The evaluation of the dispersion stability of RCMNs according to the changes of T_2_ relaxation rates j), zeta potentials k), and particle sizes l) in 2 weeks.

With amino‐terminal nucleophiles attacking C—F bonds, ROS‐response AT (capturing ROS) and hydrophilic THAM (improving the aqueous dispersity) were integrated onto FCNs carriers, and eventually, the anticipated RCMNs were fabricated (Figure 1a). RCMN‐1, RCMN‐2, and RCMN‐3 corresponded to the grafted products of FCN‐1, FCN‐2, and FCN‐3, respectively (**Figure** **2**h). After carrying out the multifunctional reaction, the detailed chemical composition of RCMNs was recorded by the FTIR spectrum (Figure S5, Supporting Information), where obvious ‐CH_3_ and ‐CH_2_ structures belonging to grafted species (AT and THAM) were observed. The enhanced peak of hydroxyl and the new peak of the C—N bonds further indicated the feasibility of attaching AT and THAM to FCNs. Meanwhile, the appearance of XPS N1s spectra suggested that AT and THAM were successfully grafted onto fluorinated carbon nanosheets (Figure S7, Supporting Information). Additionally, the XPS C1s spectra demonstrated the changes in the proportion of the different components (Figure 1d,g; Figure S1b–e, Supporting Information). When FCNs were reacted with AT and THAM, a portion of C—F bonds were consumed as a result of their involvement in the reaction, resulting in decreased fluorine content. Compared with the negligible proportion of isolated C—F bonds in FCNs, there were more isolated C—F bonds in RCMNs (Figure S6a–f, Supporting Information), which could be ascribed to the influence of steric hindrance and reductive defluorination during the reaction. The derivative thermogravimetric (DTG) curve (Figure 1e) provided further evidence of chemical modification. FCNs showed the weight loss only at ≈200 °C, corresponding to the decomposition of oxygen‐ and fluorine‐containing groups. Multifunctional RCMNs showed two main loss peaks, appearing at ≈200 °C and 300 °C, which were attributed to a weight loss of the original FCNs’ groups and degradation of grafted species, respectively. XRD patterns (Figure 1f) showed that the peak position of chemically modified FCNs shifted dramatically to a lower angle compared with FCNs. It indicated that the corresponding interlayer distance increased owing to successful chemical intercalation. Raman spectra also corroborated the result that with the introduction of sp^3^‐hybridized carbon, the *I*_D_/*I*_G_ ratio of RCMNs significantly increased (Figure S8, Supporting Information).

The morphology and size investigations of RCMNs were obtained using scanning electron microscopy (SEM) and atomic force microscopy (AFM). RCMNs presented a clear flake shape (Figure 2a,c) with an average particle size of 300 nm (Figure 2b) and thickness of up to 7.2 nm (Figure 2d). With the introduction of multiple hydroxyl groups (THAM), FCNs were altered from being hydrophobic to being water‐dispersible (Figure 2e; Figure S4, Supporting Information). The dispersion stability of the nanoprobe RCMNs was specifically investigated in phosphate‐buffered saline (PBS) by measuring the diameter and zeta potential changes with dynamic light scattering (DLS). Both diameters and zeta potential virtually maintained constant values in 2 weeks (Figure 2k,l). Besides, no significant difference was observed in the MRI relaxation properties of the material before and after 2 weeks (Figure 2j). Therefore, the RCMNs had a favorable stability, amenable to subsequent biological tests.

Moreover, EPR signals of different RCMNs in PBS solution (Figure 2i) presented a typical three‐line hyperfine structure of materials bearing TEMPO moieties,^[^ ^54^, ^55^ ^]^ making it possible to scavenge ROS. Furthermore, to confirm the contribution of TEMPO moieties to the EPR signal of RCMNs, a control probe without ROS‐response moieties (RCMN‐C1) was prepared by replacing AT with 2,2,6,6‐tetramethyl piperidine‐4‐amine (ATP) during the reaction process (Figure 2h). As expected, there was no obvious EPR signal from the control nanoprobe RCMN‐C1 (Figure 2f).

To test whether RCMNs could effectively improve contrast enhancement in MRI, MRI phantoms of RCMNs’ dispersions in PBS were scanned using a 7.0 T MRI instrument (Figure S11a, Supporting Information). As shown in Figure 2f, the multifunctional nanoprobe RCMNs exhibited good contrast enhancement in T_2_ mode. The values of T_2_‐weighted signals gradually dropped as the RCMNs concentration increased. Furtherly, quantitative relationships between fluorine (F) concentration (see Table S3, Supporting Information for details) and transversal relaxation time (*T*_2_) were identified in combination with energy‐dispersive spectroscopy (EDS) data (Figures S9 and S10, Supporting Information). It showed a linear increase in relaxation rate (1/*T*_2_) with the increase of F concentration (Figure 2g). According to the equation $1/T_{2}=1/T_{2}^{0}\;+r_{2}\left[F\right]$ (where $1/T_{2}^{0}$ is the inherent relaxivity rate of pure PBS and [F] is the fluorine concentration in CAs), the relaxivity value (*r*_2_) of RCmN‐1 reached 25.950 mM^−1^ s^−1^, with the same order of magnitude as that in the available literature.^[^ ^56^ ^]^ The relatively lower relaxivity values of RCMN‐2 and RCMN‐3 might result from the difference of C—F bonds’ distribution. RCMN‐2 and RCMN‐3 originated from FCNs with higher fluorine contents, but more residual C—F bonds existed between the nanosheets, resulting in a weaker interaction with water protons. Besides, considering the presence of fluorine in the material (Figure S14, Supporting Information), the feasibility 19F‐MRI imaging was also considered. Subsequently, a weak ^19^F signal was obtained in the pre‐scan (Figure S15, Supporting Information). However, it was difficult to further obtain the ideal ^19^F image due to the detection accuracy of devices and solubility of the materials (Figure S16, Supporting Information). Therefore, RCMN‐1 was regarded as the T2 probe to continue follow‐up biological tests.

### 2.2 Intracellular Performance of RCMN‐1

**Figure 3:**
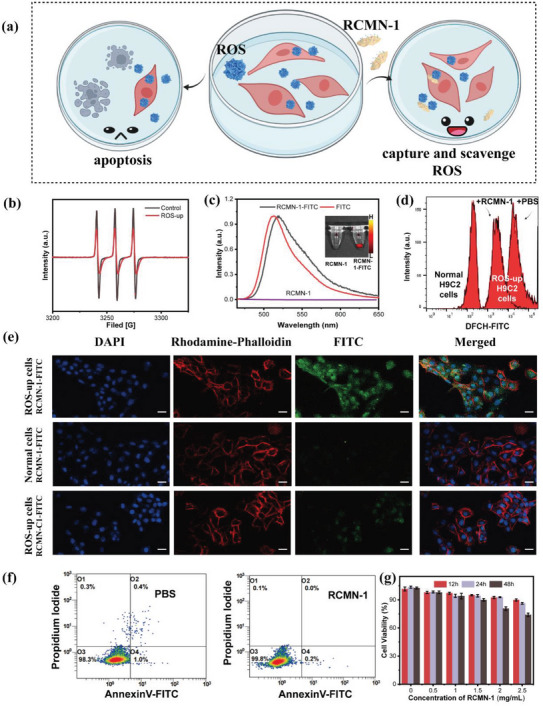
a) Illustration of RCMNs’ capturing and scavenging ROS at the cellular level. b) EPR scanning validation of RCMN‐1 capture ability in cell levels (ROS‐up represents a higher level of ROS in cells). c) Fluorescence profiles of RCMN‐1 before and after being loaded with FITC. d) ROS flow cytometry results of H9C2 cells after different treatment. e) Confocal images of cells after ingestion of probes, scale bar: 20 µm. Blue fluorescence: nuclei stained with DAPI, red fluorescence: cytoskeleton stained with Rhodamine‐Phalloidin, green fluorescence: the nanoprobes RCMNs loaded with FITC. f) The apoptosis of H9C2 cardiomyocytes induced by RCMN‐1 (100 mm based on F) for 24 h was evaluated by using flow cytometry. g) The cytotoxicity of RCMN‐1 nanoprobe tested on H9C2 cells with a CCK‐8 kit.

Inspired by the prominent ability to capture and scavenge ROS of TEMPO and its derivatives,^[^ ^38^, ^39^, ^40^, ^41^, ^42^, ^43^ ^]^ we first investigated the effects of RCMN‐1 on the ROS in cells level to assess its feasibility for MRI of ROS. ROS assay kits were used to increase the intracellular ROS content and then RCMN‐1 was added into the H9C2 cardiomyocytes. More and more TEMPO moieties were consumed due to the interaction between RCMN‐1 and ROS, theoretically resulting in a significant decrease of the EPR signal intensity. As might be expected, variations of EPR signal intensity (**Figure** **3**b) and quantitative calculation of radical concentrations (Figure S17, Supporting Information) indicated that RCMN‐1 could capture and scavenge free radicals in cells, which provides a basis to carry out subsequent experiments. To clarify the effect of ROS scavenging on *T*_2_ relaxation, we tested the *T*_2_ relaxation time of RCMN‐1 after adding ROS or PBS. The results demonstrated that whether the unpaired electrons of TEMPO were quenched by ROS or not, hardly affected the *T*_2_ relaxation of RCMN‐1 (Figure S18, Supporting Information). Additionally, given that TEMPO moieties could scavenge ROS, the therapeutic potential of RCMN‐1 was further evaluated. The above ROS assay kit for increasing the amount of intracellular ROS in H9C2 cells was used to simulate the state of elevated oxidative stress in cardiomyocytes. Additionally, the intracellular ROS content was monitored using flow cytometry and the results revealed that the ROS levels in cell samples periodically incubated with RCMN‐1 significantly decreased (Figure 3d). These results indirectly illustrated that RCMN‐1 might serve as a probe for oxidative stress management (Figure 3a).

To collect information on cell phagocytosis of the nanoprobes, we first loaded an appropriate amount of fluorescein isothiocyanate (FITC) onto RCMN‐1 to obtain RCMN‐1‐FITC. The fluorescence spectrum of the FITC‐modified RCMN‐1 sample was obtained using an ultraviolet and visible (UV–vis) spectrophotometer, and the maximum emission wavelength was obtained at 520 nm, which proved that the FITC was successfully connected (Figure 3c). Further evaluation results demonstrated that loaded FITC had little effect on MRI relaxation property (Figure S19a–c, Supporting Information) of RCMN‐1 and maintained its ROS capturing ability (Figure S19d, Supporting Information). Subsequently, the cells were incubated with RCMN‐1‐FITC, subsequently stained with Rhodamine‐Phalloidin and 4',6‐diamidino‐2‐phenylindole (DAPI), and then examined by using confocal imaging. As shown in Figure 3e, discrete green fluorescent dots (FITC) were distributed in the cytoplasm between the red cytoskeleton (Rhodamine‐Phalloidin) and the blue nucleus (DAPI), which demonstrated that the nanoprobe was taken up by the H9C2 cells. With the increase of ROS levels in cells, more RCMN‐1 probes were internalized. Furthermore, the uptake of RCMN‐1 with ROS‐response ability by the H9C2 cells was much greater than that of RCMN‐C1. Theoretically, the captured ROS serves as the functional groups of RCMN‐1 and enhances the interaction between the nanoprobe and cells, which legitimately increases the retention of nanoprobes in cells and promotes the enrichment of the ROS functionalized RCMN‐1 (RCMNs‐ROS) in the aggregation sites of ROS. The enrichment behavior is an important prerequisite for the following early diagnosis of oxidative stress by MRI imaging with the assistance of RCMN‐1.

In addition, cell flow cytometry results (Figures 3f) showed a low level of apoptosis induced by RCMN‐1 in H9C2 cardiomyocytes, which preliminarily confirmed the biocompatibility of the nanoprobe. Furthermore, H9C2 cells were also used to evaluate the RCMN‐1 cytotoxicity. Results of a cell counting kit‐8 (CCK‐8) assay (Figure 3g) showed the cell viability of all cell samples was >85%, indicating that RCMN‐1 had almost no cytotoxicity and hardly affected cell proliferation.

### 2.3 RCMN‐1 for Early Diagnosis and Therapy of DCM

Thanks to the in vitro performance of RCMN‐1, we furtherly explored its diagnostic and therapeutic effects in the DCM mice model. Leptin receptor knockout mice (db mice) served as research models of diabetes. The mice were fed with high‐fat and high‐sugar diets. As shown in Figure S20e (Supporting Information), their body weight rapidly increased. Moreover, the results of ELISA experiments indicated that db mice showed a significant change in several oxidative stress indicator with decreased antioxidant enzymes (SOD: superoxide dismutase, Figure S20b (Supporting Information); CAT: catalase, Figure S20c, Supporting Information) and increased lipid peroxidation products (MDA: malondialdehyde, Figure S20a, Supporting Information) compared to normal mice. ROS staining of heart slices also showed an increase of the ROS contents in db mice (Figure S20d, Supporting Information). Unlike the normal mice, blood glucose levels in db mice continued to increase until 8 weeks of age and remained relatively stable hereafter (Figure S20e, Supporting Information). Considering the above trend, follow‐up animal experiments started from 8 weeks of age.

Given that RCMN‐1 could capture ROS in cells, EPR scans on mice heart samples were further performed using RCMN‐1 as a trapper to confirm its sensitivity to ROS at the tissue level. After capturing, the EPR signal intensity of the db mice sample decreased significantly, because more TEMPO moieties were consumed to capture excessive ROS in db mice hearts, compared with the normal mice (Figure S20g, Supporting Information). This also further indicated that RCMN‐1 had a good response to ROS in the heart. Moreover, the subsequent experiment demonstrated that RCMN‐1 could maintain the ROS scavenging ability for a long time after injection into the mouse body (Figure S21, Supporting Information).

**Figure 4:**
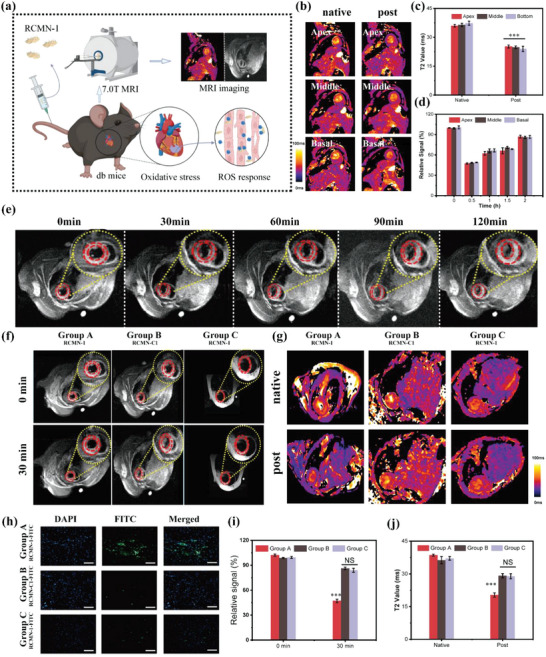
a) Schematic illustration of RCMN‐1 for early diagnosis of DCM. T_2_ maps b) and T_2_ values c) before and after probe injection (The “Apex”, “Middle” and “Basal” represent the apical, middle and basal slices of the heart, respectively.). Cardiac MRI black blood images e) and quantitative analysis d) at different time points after intravenous injection of RCMN‐1 into db mice. Cardiac MRI T_2_ black blood images f) and relative quantitative calculation i), T_2_ maps g), and T_2_ values j) of the three groups of mice before and 30 min after injection (Group A: db mice injected with RCMN‐1, group B: db mice injected with the contrast probe RCMN‐1 without ROS‐response properties, and group C: normal mice injected with RCMN‐1). h) Confocal images of heart slices of different groups of mice after administration, scale bar: 100 µm. Blue fluorescence: nuclei stained with DAPI, green fluorescence: the nanoprobes RCMNs loaded with FITC. Data in (c), (i), and (j) represent mean values ± standard deviations (SD), *n* = 5. Statistical differences were determined by unpaired Student's *t*‐test. NS means no significant difference. ***p < 0.001.

To verify the diagnostic performance of RCMN‐1 for ROS in vivo, RCMN‐1 was administered by tail vein injection into db mice and performed MRI imaging (**Figure** **4**a, see the details in Figure S11, Supporting Information). The remarkable change of the *T*_2_ mapping values before and after administration indicated that RCMN‐1 was a sensitive MRI T_2_ probe (Figure 4b,c). As shown in Figure 4d,e, the signal value of T_2_ black blood reached a minimum after 30 min administration of RCMN‐1, which decreased by ≈50%. Furthermore, the aggregation of RCMN‐1 nanoprobes in the heart site was also confirmed by fluorescence imaging with the assistance of RCMN‐1 loaded by FITC (Figure S22b,c, Supporting Information). With the blood circulation and metabolism, the T_2_ black‐blood value then gradually returned to the normal state. Subsequently, the results of plasma drug concentration measurement indicated that the peak time of the plasma drug concentration was ≈20 min (Figure S22a, Supporting Information), which was basically consistent with the aforementioned findings of MRI scans. Therefore, the optimal scan time for MRI after RCMN‐1 injection was approximately determined to be at half an hour after administration.

Motivated by the above results, whether the RCMN‐1 could effectively diagnose the oxidative stress of DCM in an early state was investigated. First, 8‐week‐old db and normal mice were randomized and injected with RCMN‐1 or RCMN‐C1 (without TEMPO modification) for MRI imaging. As expected, the T_2_ signal intensity in myocardial sites of db mice remarkably decreased by 50% at 30 min post‐injection of RCMN‐1 (group A). In contrast, that of the db mice injected with RCMN‐C1 (group B) and normal mice injected with RCMN‐1 (group C) only decreased by 20% and 17%, respectively (Figure 4f,i). The corresponding *T*_2_ value of group A also changed greatly (Figure 4g,j). Moreover, the pathological fluorescent staining results of heart sections and the quantified fluorine contents of mice hearts via ion chromatography (Figure S23, Supporting Information) also showed that more probes were distributed in the mice hearts of group A (Figure 4h). Obviously, there were significant statistical differences among three groups of mice. In groups A and B, both RCMN‐1 and RCMN‐C1 would retain a part in db mice hearts with blood circulation; however, RCMN‐1 with free‐radical trapping ability could interact with excessively produced ROS, contributing to the efficient accumulation of RCMN‐1 nanoprobes in the heart. When referring to the comparisons between groups A and C, the ROS content in db mice was much higher than that in normal mice, which enriched more RCMN‐1 in db mice hearts and realized a greater decrease of T_2_ signal. When MRI imaging was also performed in 12‐ and 16‐week‐old mice, changes of myocardial signal were similar to those in 8‐week‐old mice (Figure S24, Supporting Information). Consequently, it was demonstrated that RCMN‐1 had excellent ROS‐response properties and could effectively diagnose oxidative stress in DCM via MRI.

As a comparison, myocardial perfusion defects were not detectable via MRI until as early as 16 weeks (Figure S25, Supporting Information), when db mice were injected with clinically used Gd‐based CAs. Unfortunately, the 16‐week‐old db mice has entered the advanced disease stage, and the myocardium begins to show irreversible fibrosis, missing the optimum time for intervention and therapy. Besides, Feraheme, an FDA approved iron nanoparticle T_2_ relaxation agent, was used for T_2_ MRI imaging of db mice and normal mice. As shown in Figure S26 (Supporting Information), myocardial signals of them decreased with the circulation of the blood, but there were no statistically significant differences in the decreasing degree, suggesting that the metal‐based T2 MRI agent (without molecular targeted property) was unable to diagnose the oxidative stress in diabetic cardiomyopathy of mice model. Additionally, there was also no significant difference in left ventricular ejection fraction (LVEF) between the normal mice and db mice by analyzing MRI cine sequence (Table S4, Supporting Information). By comparison, the novel RCMN‐1 probes not only are suitable for the specific identification of ROS in DCM mice models, but also can bring the detection of DCM‐associated pathological changes forwards 8 weeks earlier. This allows more time for follow‐up treatment, which avoids irreversible lesions in heart. Moreover, it is worth expecting that the RCMNs probes and the corresponding early diagnosis strategy are extended to the other ROS related diseases.

**Figure 5:**
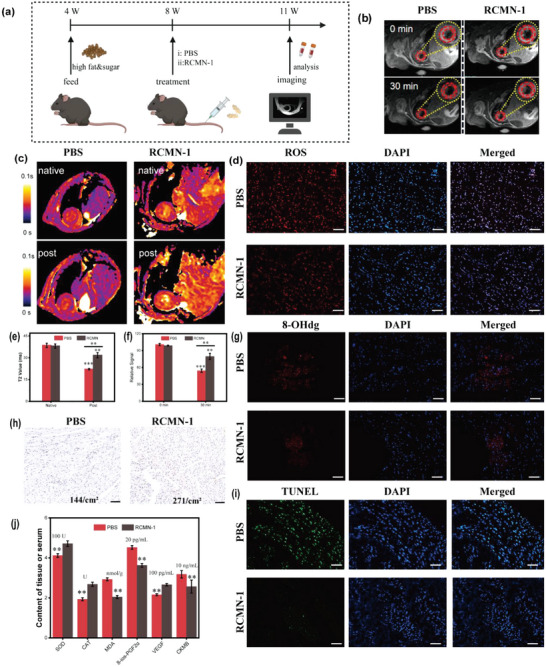
a) Schematic illustration of RCMN for ROS scavenging in vivo. MRI black blood images b) and myocardial signal e), T_2_ maps c) and values f) before and after injection of different reagents (PBS or RCMN‐1, “native” and “post” represent MRI T_2_ maps before and after injection of the material, respectively.). ROS d), 8‐OHdG g), and CD31 h) of mice heart sections after administration of different treatment regents (PBS or RCMN‐1), scale bar: 100 µm. i) H&E staining of different organs in mice, scale bar: 100 µm. j) Changes of oxidative stress indicators (lipid peroxidation products: malondialdehyde, MDA; 8‐iso‐prostaglandinF_2*α*_, 8‐ios‐PGF_2*α*_, and antioxidant enzymes: catalase, CAT; superoxide dismutase, SOD) and cardiac function indicators (vascular endothelial growth factor, VEGF and Creatine Kinase‐MB, CKMB). Data in (e) and (f) represent mean values ± SD, *n* = 5. Statistical differences were determined by unpaired Student's *t*‐test. NS means no significant difference. ***p < 0.001.

Furtherly, the therapeutic efficacy of RCMN‐1 nanoprobes was investigated. we randomly divided 12 8‐week‐old db mice into two groups. The mice were administered an appropriate amount of PBS (the control group) or RCMN‐1 (4 µL g^−1^) twice a day, respectively (**Figure** **5**a). After continuous administration for 3 weeks, MRI scans were performed and then the mice were sacrificed to collect their hearts for EPR scanning, oxidative stress biochemical detection, and pathological staining analysis, respectively. Results of MRI (Figure 5b,c,e,f), EPR (Figure S27, Supporting Information), ROS staining (Figure 5d), 8‐hydroxy‐2′‐deoxyguanosine (8‐OHdG) staining (Figure 5g) and oxidative stress indicators of myocardial tissue and serum (Figure 5j) all suggested that the myocardial ROS contents of the mice in the treatment group were lower than those in the control group, which was based on the fact that TEMPO moieties of RCMN‐1 consumed a part of ROS and thus relieved the oxidative stress in the body. Furthermore, the staining results of CD31 (Figure 5h) indicated that the microvessel density of the treatment group was higher than that of the control group. H&E staining (Figure S28, Supporting Information) showed that the hearts of the treatment group had less inflammatory cell infiltration. On the basis of the staining results TUNEL (Figure 5i) in pathological sections, less apoptosis was measured in cardiomyocytes isolated from the hearts of treatment‐group mice. Overall, the novel nanoprobe RCMN‐1 relieved oxidative stress by capturing and scavenging ROS in the body, improved the microenvironment of cardiomyocytes, and protected cardiomyocytes to some extent, which effectively suppresses the pathological progression of DCM in mice models.

### 2.4 Biosafety Evaluation of RCMN‐1

To assess the toxicity of RCMN‐1 in vivo, a whole blood analysis and histological examination were conducted. First, the RCMN‐1 nanoprobes were intravenously injected into healthy female BALB/c mice via the caudal vein at a dose of 4 µL g^−1^, while PBS was administered to the control‐group mice at the same dose. Afterwards, all mice were sacrificed to collect their blood for whole blood analysis (Figure S29, Supporting Information) and serum biochemical analysis (Figure S30, Supporting Information). The levels of white blood cells, lymphocytes, monocytes, red blood cells, hemoglobin, and platelets were all in the normal ranges (Figure S29, Supporting Information), which indicated that there was no acute toxicity after injection of RCMN‐1. Moreover, the results of serum biochemical analysis also suggested that indicators of liver damage (alanine aminotransferase: ALT and aspartate aminotransferase: AST), kidney damage (blood urea nitrogen: UREA and creatinine: CREA‐K), and myocardium damage (lactate dehydrogenase: LDH) were all within the normal ranges (Figure S30, Supporting Information). To further determine the effect of periodic injections of RCMN‐1 on the mice, the mice were euthanized and their organ tissues were prepared for H&E (Figure S31a, Supporting Information) and TUNEL (Figure S31b, Supporting Information) staining after MRI imaging. The histological examination revealed no apparent organ damage to the liver, heart, kidney, spleen, or lung tissues, which indicated an appreciable safety (Figure S31a, Supporting Information). TUNEL staining of organ tissues demonstrated that administration of PBS or RCMN‐1 did not exacerbate the cell apoptosis (Figure S31b, Supporting Information). The body weight of mice treated with RCMN‐1 had no significant difference compared with those of the control group (Figure S32, Supporting Information). In summary, these results demonstrated the appreciable safety of the RCMN‐1 nanoprobes. Furthermore, the biodistribution of RCMN‐1 was also preliminarily evaluated by fluorescence imaging (Figure S22b,c, Supporting Information). It was shown that fluorescence signals of materials were clearly observed in the heart, lungs, liver, and kidneys at 30 min after injection. In contrast, corresponding signals were mainly distributed in the liver and kidneys at 24 h post‐injection, consistent with the previous report.^[^ ^57^ ^]^ Additionally, the fluorine contents of different organs of the mice at 0.5/24 h post‐injection were also quantified (Figure S33, Supporting Information), so as to describe in vivo distribution of the probe more adequately. Similarly, the probe mainly accumulated in the heart, liver, and kidney at 0.5 h. After 24 h, the concentrations of fluorine in the kidney and liver decreased significantly. It can be inferred that RCMN‐1 was metabolized through the liver and kidney.

## 3 Conclusion

We have developed a novel, metal‐free ROS‐sensitive T_2_‐weighted MRI nanoprobe, RCMN‐1, for the early diagnosis and therapy of DCM by using the FCNs as functional carriers. The introduction of multiple hydroxyl groups onto their surface greatly improved the water dispersibility of FCNs, and the coexistence of TEMPO moieties and C‐F bonds successfully combined the ROS‐sensitive property and the high resolution and the anatomical information deriving from MRI. When exposed to ROS‐rich microenvironment of the injured hearts, RCMN‐1 was actually functionalized by the captured ROS groups. This promoted a brief enrichment of the material in the myocardium and thus caused a variation of the corresponding MRI signal, which realized the in vivo MRI imaging of ROS. Furthermore, the TEMPO moieties could scavenge ROS to alleviate oxidative stress and create a good microenvironment for the cardiac regeneration, which induced a significantly lower oxidative stress index of treated mice than that of the control mice. Overall, the metal‐free nanoprobe RCMN‐1 could realize the earlier diagnosis and therapeutic potential of DCM, and thus further expand the biological applications of the carbon‐based materials. Moreover, it is worth expecting that the corresponding strategy for early diagnosis and therapy could be extended to the other ROS‐related diseases, such as myocardial infarction, stroke, and tumor.

## 4 Experimental Section

The full experimental details and characterization methods can be found in the Supporting Information. All animals used in this study were in accordance with protocols outlined and approved by the Institutional Animal Care and Use Committee of West China Second Hospital, Sichuan University (WCSUH21‐2021‐026).

## Conflict of Interest

The authors declare no conflict of interest.

## Author Contributions

Z.N. and K.Z. contributed equally to this work. The manuscript was written through the contributions of all authors. All authors have given approval to the final version of the manuscript.

## Supporting information

Supporting information

Supporting information

## Data Availability

The data that support the findings of this study are available in the supplementary material of this article.
