# Supplementary material for: A Multifunctional Integrated Metal‐Free MRI Agent for Early Diagnosis of Oxidative Stress in a Mouse Model of Diabetic Cardiomyopathy
Source: Adv Sci (Weinh). 2023 Jan 3;10(7):2206171. doi: 10.1002/advs.202206171 (PMC9982554; doi:10.1002/advs.202206171)
Supplement: Supplementary file 1 — Supporting information [file ADVS-10-2206171-s001.pdf]

Supporting Information

A Multifunctional Integrated Metal-Free MRI Probe for Early Diagnosis of Oxidative Stress in Diabetic Cardiomyopathy

Zhuang Nie,[a1] Kun Zhang,[b1] Xinyu Chen,[a] Jingxin Wang,[b] Huile Gao,[c] Bingwen Zheng,[d] Qihong Wu,[b] Yingkun Guo,[b] Xiangyang Liu, [a]* Xu Wang[a]**

[1] Zhuang Nie and Kun Zhang contributed equally to this work (co-first author).

[a] College of Polymer Science and Engineering, State Key Laboratory of Polymer Material and Engineering, Sichuan University, Chengdu 610065, P. R. China.

[b] Department of Radiology, Key Laboratory of Birth Defects and Related Diseases of Women and Children of Ministry of Education, West China Second University Hospital, Sichuan University, 20# South Renmin Road, Chengdu, Sichuan 610041, China.

[c] Key Laboratory of Drug-Targeting and Drug Delivery System of the Education Ministry, Sichuan Engineering Laboratory for Plant-Sourced Drug and Sichuan Research Center for Drug Precision Industrial Technology, West China School of Pharmacy, Sichuan University, Chengdu 610064, China.

[d] Time Medical Ltd., Hong Kong Science & Technology Park, Hong Kong 999077, China.

*Corresponding authors

[a] Tel.: +86 28 85403948. Fax: +86 28 85405138.

E-mail address: wangxu@scu.edu.cn (Xu Wang), lxy6912@sina.com (Xiangyang Liu)

[b] Tel.: +86 28 88570307. Fax: +86 28 88570445.

E-mail address: gykpanda@163.com (Yingkun Guo)

Experimental Section

1. Preparation of ROS-response contrast-enhanced MRI nanoprobe (RCMN)

1. 1. Materials

Graphene oxide (GO, analytical grade) was obtained from Suzhou TANFENG graphene Tech Co., Ltd. Graphene was purchased from the Sixth Elementary (Changzhou) Materials Technology Co., Ltd. The mixture gas of F_2/N_2 (volume ratio=1:4) with a purity of 99.99% was purchased from Do-Fluoride Chemicals Co., Ltd. 2,2,6,6-Tetramethyl-4-Amino-1-Piperidinyloxy (4-Amino-Tempo, AT), 2,2,6,6-Tetramethylpiperidin-4-amine (ATP), and tris(hydroxymethyl)aminomethane (THAM) were acquired from Adamas Reagent, Ltd. Ethanol with commercially analytical grade and deionized water was purchased from Kelong Chemical Reagent Co., Ltd (Chengdu), and used without further purification.

1.2. Preparation of fluorinated carbon nanosheets (FCNs)

300 mg graphene oxide was placed in a closed stainless steel (SUS316) chamber (20 L) equipped with a vacuum line. Firstly, we exchanged internal air and moisture with N_2 three times to exclude their influence for the fluorination process. And then, 80 kPa mixed gas (F_2/N_2) was added into the reactor at room temperature (RT), keeping for a certain amount of time to guarantee GO to react with F_2 by sufficient contact. Furthermore, taking account of paramagnetic behavior and hydrophobicity of

FCNs, we prepared three kinds of FCNs (FCN-1, FCN-2 and FCN-3) with different fluorination degree by adjusting fluorination process. After reacting for an hour at RT, the residuary F_2 and generated gases like HF were completely absorbed by absorption tower, and FCN-1 was finally obtained. In contrast, FCN-2 and FCN-3 were fabricated by fluorinating GO two times. After absorption of the redundant gas, subsequent 80 kPa F_2/N_2 mixed gas was introduced into the chamber again, keeping for 4 h or 9 h. The corresponding products were denoted as FCN-2 and FCN-3 respectively. Additionally, the FCNs with a higher fluorination degree (h-FCN) were prepared by increasing the reaction temperature from RT to 80 °C. After reacting for an hour at 80 °C, the residuary F_2 and by-products were disposed according to the above-mentioned procedure, and h-FCN was finally obtained. In addition, Graphene was treated with the mixed gas to fabricate three different fluorinated graphene (FG) by adjusting the fluorination process, and the corresponding products were denoted as FG-1, FG-2, FG-3, respectively.

1.3. Preparation of RCMNs

80 mg FCNs (FCN-1, FCN-2 or FCN-3) was dispersed in ethanol (50 mL), and then sonicated for 30 min. Subsequently, the mixture was degassed under continuous argon gas flow. 4-Amino-Tempo and tris(hydroxymethyl)aminomethane (predissolved in deionized water) was separately dissolved in ethanol (15 mL) by different proportions (details see the information in table S1), followed by analogous

degassing. Next, reaction system is cooled to about -45 ℃, and the AT solution was added dropwise. After stirring for 1 h, another solution of THAM was added to schlenk bottle gradually, and the reaction was stirred at -45 ℃ for 1 h again. Afterwards, the functionalized FCNs (fFCNs), namely RCMNs, were separated by high-speed centrifugation, and further purified with alcohol washing more than once until there was no obvious characteristic absorption peak of AT and THAM in the UV-vis absorption spectra of the supernatant. In addition, to eliminate materials with a larger size, centrifugation ran at a low speed (1000 rpm) after repeated washing. Eventually, the purified fFCNs was dried overnight at RT in vacuum. RCMN-1, RCMN-2 and RCMN-3 respectively corresponded to the functionalized products of FCN-1, FCN-2 and FCN-3. For comparison to free radical-responsive RCMN and excluding electron paramagnetic resonance (EPR) signal interference of potential radicals on graphene sheets, a control probe RCMN-C1 without free radical-responsive moiety was prepared by replacing AT with ATP during the reaction process (details see the information in Table S1).

Table S1. Covalent multifunction of various FCNs

	RCMN-1	RCMN-C1	RCMN-2	RCMN-3
FCNs	FCN-1	FCN-1	FCN-2	FCN-3
	51.4	---	51.4	51.4

AT<br>(%, mass ratio of AT/FCNs)				
ATP<br>(%, mass ratio of ATP/FCNs)	---	46.9	---	---
THAM<br>(%, mass ratio of THAM/FCNs)	34.3	34.3	42.4	42.4

2. Material characterization

Fourier-transform infrared spectrum (FTIR) was performed utilizing a Nicolet 560 FTIR instrument in the wavenumber range between 500 and 4000 cm^{-1}. X-ray diffraction (XRD) patterns were performed on an Ultima IV powder diffractometer (Rigaku Corporation) with a Cu Kα radiation within the 2θ = 5-90° range. The surface chemical composition and valence band spectrum of as-obtained products was examined by X-ray photoelectron spectroscopy (XPS) on a ESCALAB Xi+ spectrometer (Thermo Fisher Scientific, US) accompanied by a monochromatic Al Ka rays (1486.6 eV) under the circumstance of 12.5 kV × 16 mA. For the core-level spectra, the pass energy and step size were set to 30 eV and 0.1 eV, respectively.

Energy dispersive X-ray spectroscopy (EDS) and mapping was conducted by Field emission scanning electron microscopy (FESEM) on Nova Nano450. UV–vis spectroscopic analysis was performed on a Shimadzu UV3600 spectrophotometer at RT. Thermal gravimetric analysis (TGA) was performed on Netzsch 209F1 with a heating rate of 10 °C min^{-1} from 35 to 800 °C under nitrogen atmosphere.

To determine the proton relaxation rates (r_2) of fFCNs, the transverse relaxation time T_2 (s) was investigated at different concentrations in 10 mM PBS buffer using a 7.0 T MRI instrument at room temperature. In brief, the fFCNs solutions (0.025, 0.05, 0.1, 1.5, 2 mg mL^{-1}) in 10 mM PBS buffer (pH=7.4; n = 3) were placed into 1.5 mL tubes. Then, the transverse relaxation time T_2 (s) were measured at room temperature. The r_2 was calculated according to the equation $r_2 = (1/T_2 - 1/T_2(0))/[F]$, where [F] represents the concentration of fFCNs, $1/T_2(0)$ (s^{-1}) is the transverse relaxation rate without paramagnetic species and $1/T_2$ (s^{-1}) is the transverse relaxation rate with CAs.

To confirm successful grafting and radical scavenging ability of RCMNs. RCMN-1, RCMN-2, RCMN-3 and RCMN-C1 were dispersed in PBS (0.4 mg/mL) respectively. EPR measurements were carried out on Bruker EPR EMX Plus (Bruker Beijing Science and Technology Ltd, USA) to capture the radical signals, operating at a frequency of approximately 9.8 GHz using a standard microwave power of 1 mW.

3. *In vitro* and *in vivo* analysis of the RCMNs nanoprobes

3.1. Materials, cell line and animals

Fluorescein isothiocyanate isomer I (FITC) was purchased from Adamas Reagent, Ltd. H&E staining kit, CD31 kit, 8-OHdg kit and TUNEL kit were obtained from Kelong Chemical Reagent Co., Ltd (Chengdu, China). The kits of superoxide dismutase (SOD), catalase (CAT), creatine kinase isoenzymes (CKMB), vascular endothelial growth factor (VEGF), 8-ios-PGEF2α and malondialdehyde (MDA) were all purchased from Nanjing Jiancheng Bioengineering Institute (Nanjing, China). The cell counting kit-8 (CCK-8) was purchased from Dojindo Laboratories (Kumamoto, Japan). The ROS assay kit was purchased from Beyotime Biotechnology (Shanghai, China). The H9C2 cells were supplied by the West China Hospital, Sichuan University (Chengdu, China) and maintained with dulbecco's modified eagle's medium (DMEM) supplemented with 10% fetal bovine serum (FBS). The FBS, DMEM, phosphate buffer saline (PBS), 4′,6-diamidino-2-phenylindole (DAPI), dihydroethidium (DHE) kit, Annexin V-FITC/PI Cell Apoptosis Detection Kit and bovine serum albumin (BSA) were purchased from Servicebio Tecchnology CO., Ltd (Wuhan, China). Gd-DTPA was purchased from GE Healthcare Life Sciences (Shanghai, China). Ferumoxytol (Feraheme) was purchased from AMAG Pharmaceuticals Inc. (Massachusetts, USA). Rhodamine-Phalloidin kit was purchased from Cytoskeleton, Inc. (Denver, USA). Isoflurane was purchased from RWD Life Science Co., Ltd. (Shenzhen, China). The BALB/c mice (female), the DCM mice models (db mice, male) and normal mice of the same strain as db mice (male) were all purchased from Chengdu Jicui Experimental Animal Co., Ltd (Chengdu, China). All

animals used in this study were in accordance with protocols outlined and approved by the Institutional Animal Care and Use Committee of West China Second Hospital, Sichuan University (WCSUH21-2021-026).

3.2. Cell uptake assay

To effectively evaluate cellular uptake of nanoparticles, RCMN-1/RCMN-C1 was loaded with generous fluorescein isothiocyanate I (FITC). Firstly, 10 mg RCMN-1/RCMN-C1 was suspended in 10 mL PBS. Next, 10 mg FITC was added to the above solution and mixed with a magnetic stirrer in the dark for 8 h. RCMN-1/RCMN-C1 loaded with FITC (RCMN-1-FITC/RCMN-C1-FITC) was separated by centrifugation, and further purified with PBS and distilled water washing to remove unbound FITC. Eventually, the product was dried overnight at RT in vacuum and preserved in the dark.

An appropriate amount of RCMN-1-FITC/RCMN-C1-FITC was added to DMEM first, and then the mixed solution was added to the petri dish with adherent cells. After 4 hours of co-incubation, staining and fluorescence photography could be carried out. For confocal imaging, the cells were fixed with 4% paraformaldehyde for 15 min, 200 μL of BSA solution of Rhodamine-Phalloidin (1:5000) was added and stained for 60 min, followed by 100 μL of DAPI for 10 min, and washed 2-3 times with PBS at the end of each operation. Confocal imaging was conducted using a Zeiss 880 confocal microscope (Carl Zeiss AG, Oberkochen, Germany) at an excitation wavelength of

405 nm and emission wavelength of 450 nm for DAPI, 460 nm (EX) and 520 nm (EM) for RCMN-1-FITC/RCMN-C1-FITC, and 540 nm (EX) and 565 nm(EM) for Rhodamine-Phalloidin respectively.

3.3. In vitro cytotoxicity test

To assess cytotoxicity, cardiomyocytes cells, H9C2 cells, were used to evaluate the cytotoxic profiles of RCMN-1 nanoprobes. H9C2 cells were incubated with RCMN-1 at various concentrations in the wells of 96-well culture plates in serum-free culture medium. Following repetitive washing with PBS to remove unbound RCMN-1, the labeled cells were further cultured in fresh cell medium for the appropriate periods of time. Finally, cell proliferation was determined by measuring cell viability with a standard CCK-8 assay, and the cell viability was measured by using a microplate reader (Cyration 3, Biotek, Vermont, USA) at 450 nm at 2 h post CCK-8 addition. Besides, whether the nanoprobes would induce cell apoptosis was also checked by flow cytometry. Briefly, the H9C2 cells were seeded in 12-well plates (10^5 cells/well) to incubate for 24 h, and then the cells were exposed to the PBS solution of RCMN-1 (100 mM based on fluorine element) for 24 h to measure the apoptosis with flow cytometry.

3.4. EPR cell experiment

In the experimental part of EPR in cells level, the ROS assay kit was added to the culture dish in which the mouse cardiomyocytes were incubated, regarded as the

experimental group, and the other group was added with the same amount of PBS as the control group. After 4 hours of incubation, trypsin digestion was added. EPR tests were performed after cell technology.

3.5. MRI imaging

Animals (mice model) were anesthetized using isoflurane (2% induced concentration and 1% maintained concentration) and scanned under a NOVA 7.0T preclinical horizontal MRI system (Time Medical Systems, Ltd.) by using a mouse heart receive coil. The CMRI protocol is shown in Figure S11c. The results of T_2 mapping and T_2 black blood sequences directly represent the corresponding signal alterations caused by the enrichment of materials.

The selection of the region of interests (ROIs) on the MRI images of the mouse heart is shown in Figure S11b. Since DCM is a diffuse lesion, we measured the average value of the whole myocardium. In order to greatly reduce the experimental error, a reference tube made by the Gd-DTPA solution was introduced in T_2 black blood scans, and the signal-to-back ratio (the ratio of myocardial signal to the standard tube signal) was used as the relative signal value for analysis. After cardiac anatomic localization (Figure S12a), the T2 black blood and T2 mapping sequences before administration were collected as the baseline. Subsequently, mice were administered via the tail vein at a dose of 4 μL/g [the ratio of fFCNs solution (2 mg/mL) to the body weight], and then the corresponding data was continuously

collected after administration. In addition, cardiac perfusion sequences (DCE) were then used to analyze the presence of perfusion defects in mice. MRI cine sequences (CINE) were acquired primarily to obtain baseline data to analyze cardiac function in mice. T_2 mapping data was fitted with the exponential model curve $S(TE) = S0\ exp.(-TE/T_2)$ from a series of raw T_2-weighted images (Figure S12b) by using the scanner software provided by MRI vendor (Time Medical Systems Co., Ltd.). Before curve fitting, noise bias correction was applied on the raw images. A simple image mask based on the intensity threshold was used in order to avoid the fitting calculation on the noise background. The R-squared map of all T_2 fittings showed a good fitting ($R^2 > 0.9$, Figure S13) in the major area of myocardium. The typical MRI experimental parameters are: the T_2 black blood and T_2 mapping sequence (FOV: 40×40 mm^2, Thickness: 1.0 mm, Gap: 0.2 mm, Flip angle: 30 deg, Averages: 3, Data matrix: 192×192), DCE sequence (FOV: 40×40 mm^2, Thickness: 1.0 mm, Gap: 0 mm, Flip angle: 38 deg, Data matrix: 128×96) and CINE (FOV: 40×40 mm^2, Thickness: 1.0 mm, Gap: 0 mm, Flip angle: 30 deg, Data matrix: 192×192).

3.6. EPR animal experiment

After the mice were euthanized, the hearts were quickly collected, and the quantitative myocardial tissue was weighed, added with an appropriate amount of RCMN-1 solution in PBS, fully homogenized, and sampled with a capillary glass tube. After measuring the liquid level, the samples were put into the scanning

chamber, followed by tuning and eventual EPR scanning (Number of counts: 3000, time constants: 20.48 ms, sweep width: 300 G, sweep time: 12.0 s).

3.7. Plasma drug concentration and biodistribution

We used a fluorescence spectrophotometer (722S, Shanghai Yitian Scientific Instruments Co., Ltd. China) to detect the plasma drug concentration. Firstly, RCMN-1-FITC solution (2 mg/mL) was injected into balb/c mice via tail vein at a dose of 4 μL/g, and blood was taken from mice at 2/5/15/30 min or 1/4/8/12/24 h after injection, followed by 3000 r and centrifugation for 15 min to obtain serum samples. After turning on the machine, the excitation wavelength of FITC (460 nm) was input, and the signal values of the serum at different time points were detected after machine zeroing with blank serum, respectively.

Mice were euthanized 30 minutes or 24 hours after tail vein injection, respectively, and the heart, liver, spleen, lungs, kidneys, brain and muscle were taken, followed by fluorescence imaging using IVIS (Lumina Series III, PerkinElmer Ltd., USA). After fluorescence imaging, individual organ and muscle samples are fixed, embedded, and sectioned, DAPI is added to stain cell nuclei, and finally a fluorescence confocal microscope is used to scan the sections and obtain information on biodistribution.

3.8. the quantification of fluorine contents in mice organs

The combustion-ion chromatography method (according to GB/T 41067-2021, China) was adopted to quantify the contents of fluorine. Specifically, the organ tissues of the mice were dried and then ground into powder. Subsequently, 50 mg of the powder was placed in a combustion bottle (an oxygen-rich atmosphere) and burned thoroughly. And then the combustion products were completely absorbed by the NaOH solution (0.02M, 10 mL) at the bottom of the bottle. Finally, the fluorine contents of the samples were tested using ion chromatography (IC, ICS600, Thermo Scientific).

3.9. In vivo toxicity evaluation

Female BALB/c mice were divided into two groups (n = 6) and intravenously administrated with RCMN-1 at the dose of 4 μL/g, while the pure PBS was intravenously injected into mice as the negative control. Then, the mice were sacrificed at predefined time points post injection, and the blood was collected for whole blood analysis and serum biochemical analysis. Meanwhile, we administered the same dose of RCMN-1-FITC to mice and euthanized them 0.5/24 h after the injection. And the main organs tissues were collected for fluorescence imaging to study bio-distribution. To further determine the effect of periodic injections of RCMN-1 on the mice, after MRI imaging was completed in the treated and control mice, the mice were euthanized and their organ tissues were prepared for H&E and

TUNEL staining. The body weight and survival of the mice were also recorded during the experiment.

3.10. Histological analysis

After MRI imaging, the mice were euthanized and their organs were harvested. The TUNEL staining and (hematoxylin and eosin) H&E staining were performed to observe vascular density, cell morphology and apoptosis. Briefly, the heart was fixed in 10% buffered formalin, embedded in paraffin and sliced into 5 μm thickness. The slices were deparaffinized in xylene, dehydrated with graded alcohols, and stained with corresponding kits. Finally, the stained slices were observation under an optical microscope (Zeiss DP80).

For immunostaining, the heart samples were incubated overnight at 4 °C with rabbit anti-mouse CD31 antibody (1:200) to identify new vessels.

For ROS staining, fresh heart tissue was frozen and sectioned, then DHE dye was added, followed by DAPI to stain the nuclei, then added anti-fluorescence quenching sealer, and finally observed using a fluorescent confocal microscope (Zeiss DP80).

For blood biochemical analysis, blood was collected by eyeball method and centrifuged to obtain serum, then CREAK, 8-ios-PGEF2α or VEGF kits were added. Subsequently, quantitative analysis was performed by microplate reader after sufficient reaction.

For biochemical analysis of cardiac tissue, after fresh hearts were taken, quantitative myocardial tissue was weighed, fully ground, and then added into SOD, CAT or MDA kits, and quantitative analysis was performed after complete reaction.

3.11. Statistical analysis

Statistical analyses were performed using SPSS (Version 23.0) and Prism (Version 7.0) software. The normality of data was checked using the Shapiro–Wilk normality test, while the homogeneity of variance assumption was evaluated using Levene's test. Data were expressed in the format of mean ± standard deviations for continuous variables. Signal variables were compared using Student's t-test, Wilcoxon test, analysis of variance, or chi-square test (Fisher's exact test), as appropriate. p value < 0.05 were considered significant.

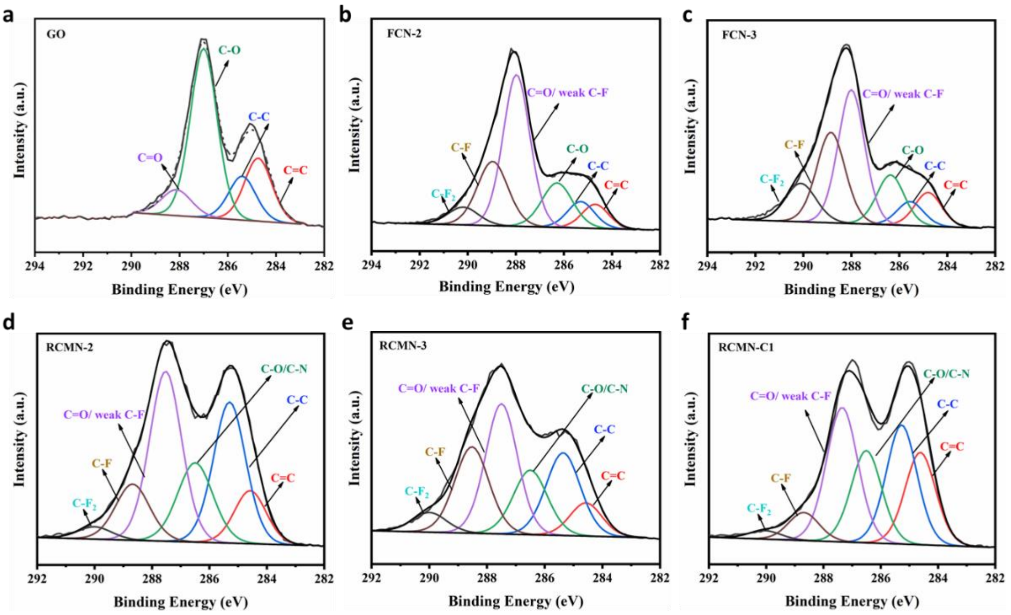

Figure S1. XPS C1s spectra of GO (a), FCN-2 (b), FCN-3(c), RCMN-2 (d), RCMN-3 (e) and RCMN-C1 (f).

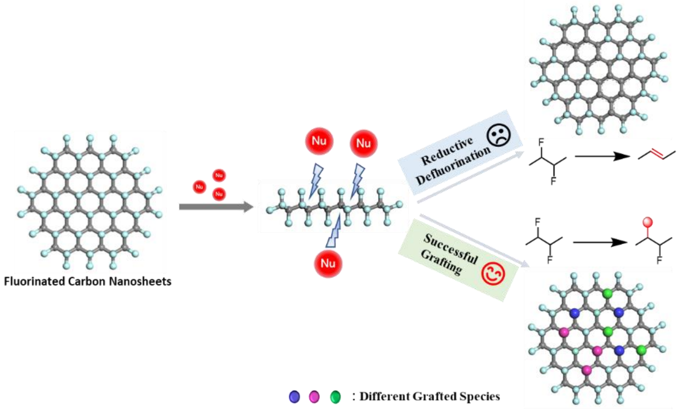

Figure S2. Schematic illustration of different reaction process of FCNs under attack of nucleophilic reagents. Successful grafting reactions are always accompanied by severe reductive defluorination, resulting in the recovery of the graphene structure and low utilization efficiency of C-F bonds.

a

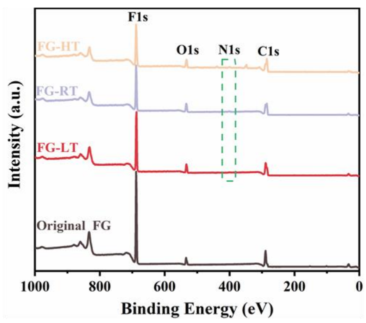

b

	XPS (at%)			
	C	N	O	F
FG	43.13	---	6.74	49.86
FG-AT-HT	52.80	2.37	10.18	34.66
FG-AT-RT	55.08	1.37	10.42	33.13
FG-AT-LT	50.01	1.12	9.23	39.64

c

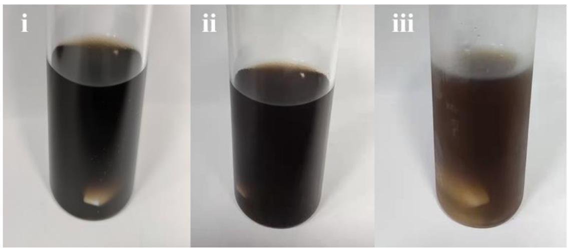

Figure S3. The reaction kinetics experiments. XPS survey spectra (a) and the corresponding atomic contents (b) of fluorinated graphene (FG) and AT grafted FG at different reaction temperature. (c) Image of reaction systems after reacting for 1h at 80 °C (i), namely HT; room temperature (RT) (ii) or -45 °C (iii), namely LT.

To quantify the grafting efficiency of fluorinated graphene at different temperature, the utilization of fluorine atoms was calculated according to the following equation:

$$\eta = \frac{0.5\mathrm{mol_N}/(\mathrm{mol_C}-4.5\mathrm{mol_N})}{\mathrm{mol_F^i}/\mathrm{mol_C^i}-\mathrm{mol_F}/(\mathrm{mol_C}-4.5\mathrm{mol_N})} \qquad \text{------ Eq. (1)}$$

where η represents the utilization ratio of C-F bonds. $\mathrm{mol_F^i}$ and $\mathrm{mol_C^i}$ represent the amounts of carbon element and fluorine element in FG. $\mathrm{mol_N}$, $\mathrm{mol_C}$ and $\mathrm{mol_F}$ represent the amounts of nitrogen element, carbon element and fluorine element in grafted products of FG (FG-AT-LT, FG-AT-RT or FG-AT-HT).

$$\eta_{\mathrm{FG-AT-HT}} = 8.434\%;$$

$$\eta_{\mathrm{FG-AT-RT}} = 2.925\%;$$

$$\eta_{\mathrm{FG-AT-LT}} = 4.535\%.$$

Table S2. The optimization of reaction conditions in functionalized FG or FCNs with grafted species AT and THAM. Additionally, the effects on water dispersion and MRI contrast enhancement were further evaluated.

		Grafted species		Water dispersion	r_2 (mM^{-1} s^{-1})
		AT	THAM		
FG	FG-1	√	×	Poor	---
		√	√	Poor	---
	FG-2	√	×	Poor	---
		√	√	Poor	---
	FG-3	√	×	Poor	---
		√	√	Poor	---
FCN	FCN-1	√	×	Poor	---
		√	√	Good	25.950
	FCN-2	√	×	Poor	---
		√	√	Good	4.441
	FCN-3	√	×	Poor	---
		√	√	Good	2.396
	h-FCN	√	×	Poor	---
		√	√	Poor	---

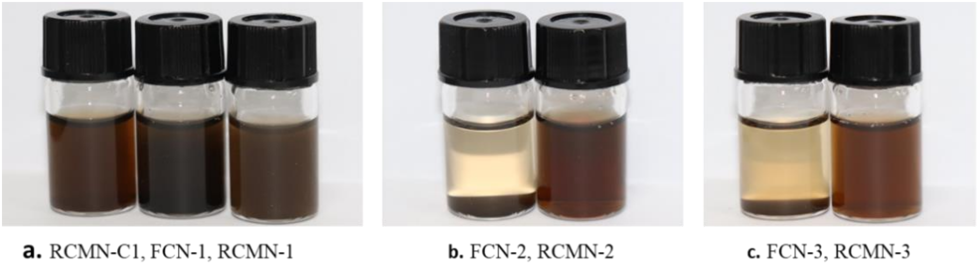

Figure S4. Digital images of FCNs and corresponding RCMNs dispersed in PBS.

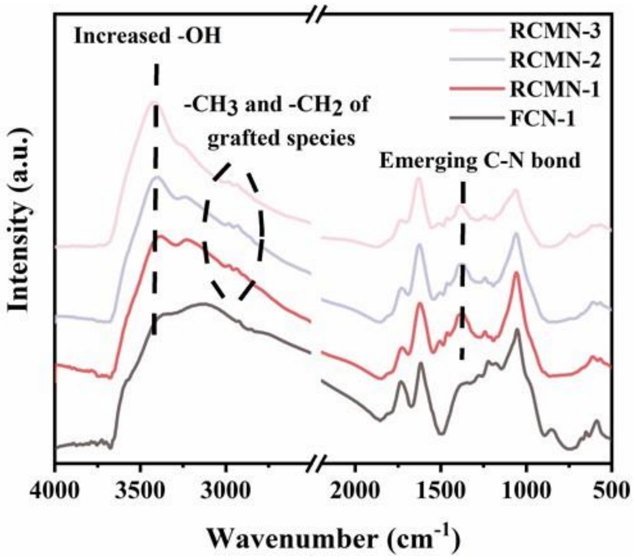

Figure S5. FTIR spectra of FCN-1 and three different RCMNs.

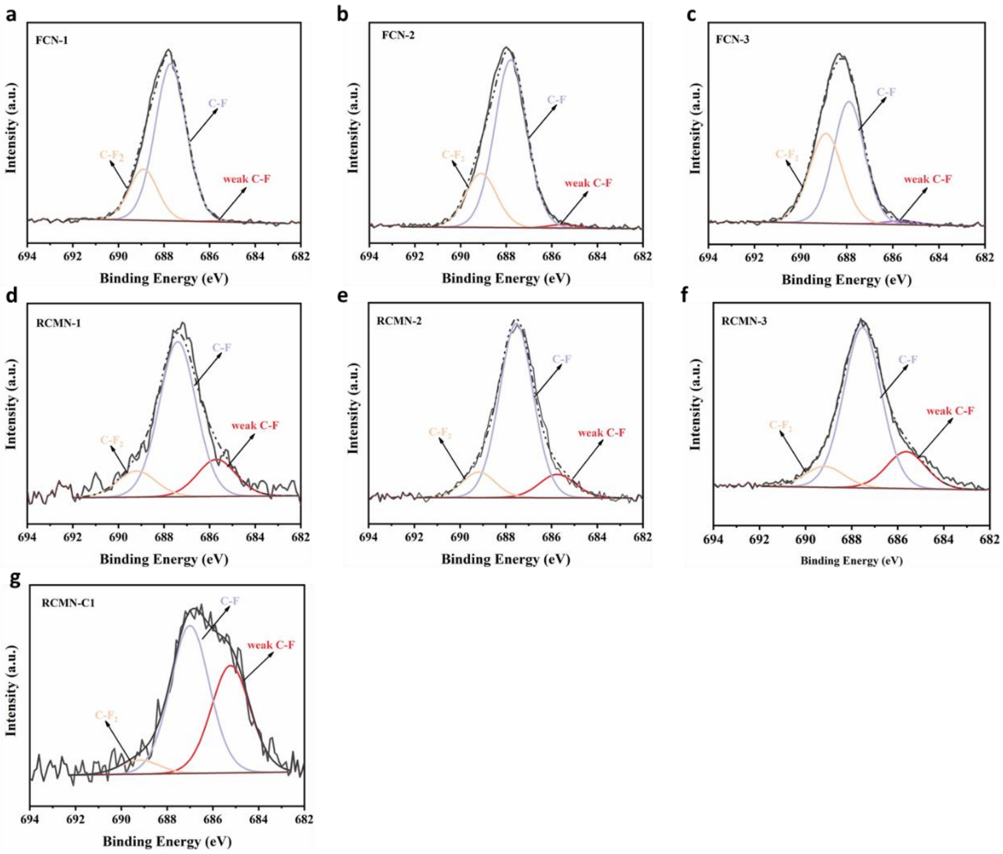

Figure S6. XPS F1s spectra of FCN-1 (a), FCN-2(b), FCN-3(c), RCMN-1 (d), RCMN-2 (e), RCMN-3 (f) and RCMN-C1 (g).

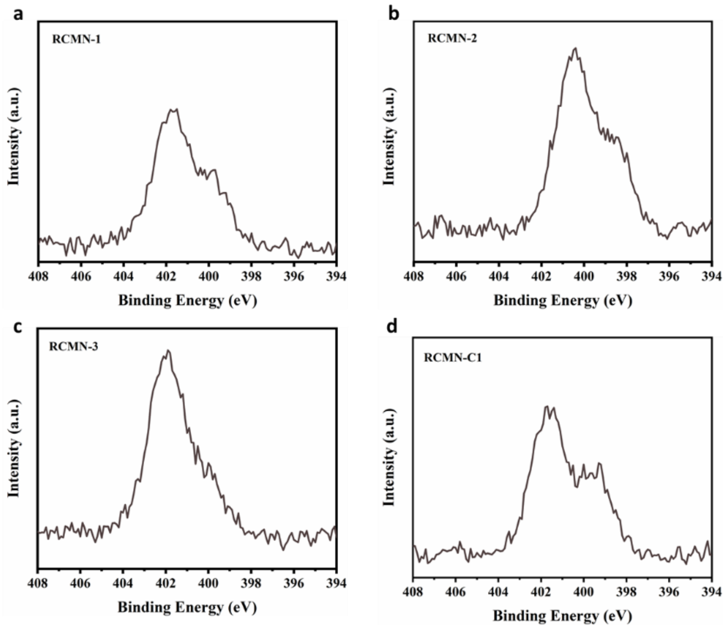

Figure S7. XPS N1s spectra of RCMN-1 (a), RCMN-2 (b), RCMN-3 (c) and RCMN-C1 (d).

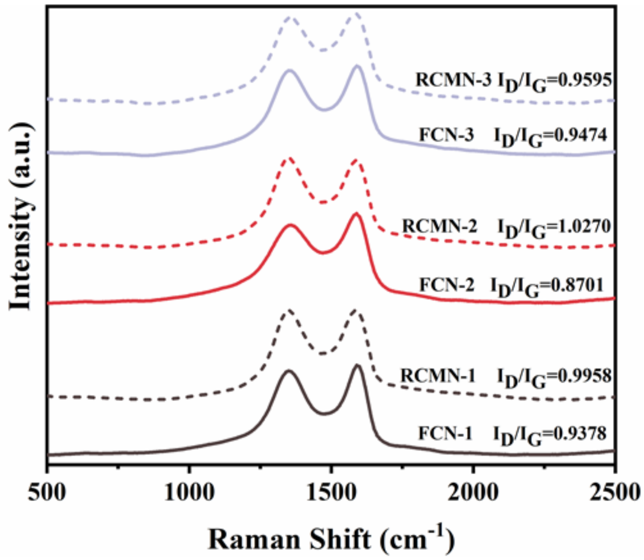

Figure S8. Raman spectra of FCNs and corresponding RCMNs.

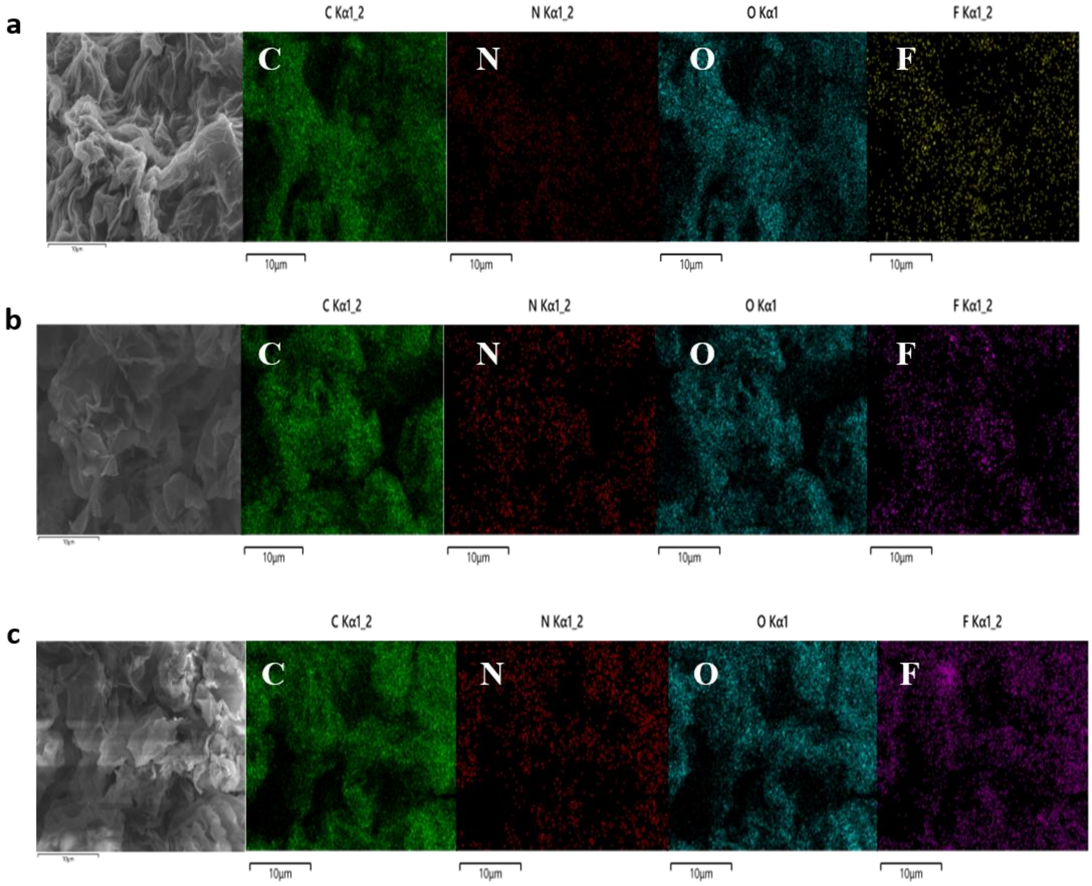

Figure S9. EDS elemental mapping of RCMN-1(a), RCMN-2 (b) and RCMN-3 (c). Scale bar: 10 μm.

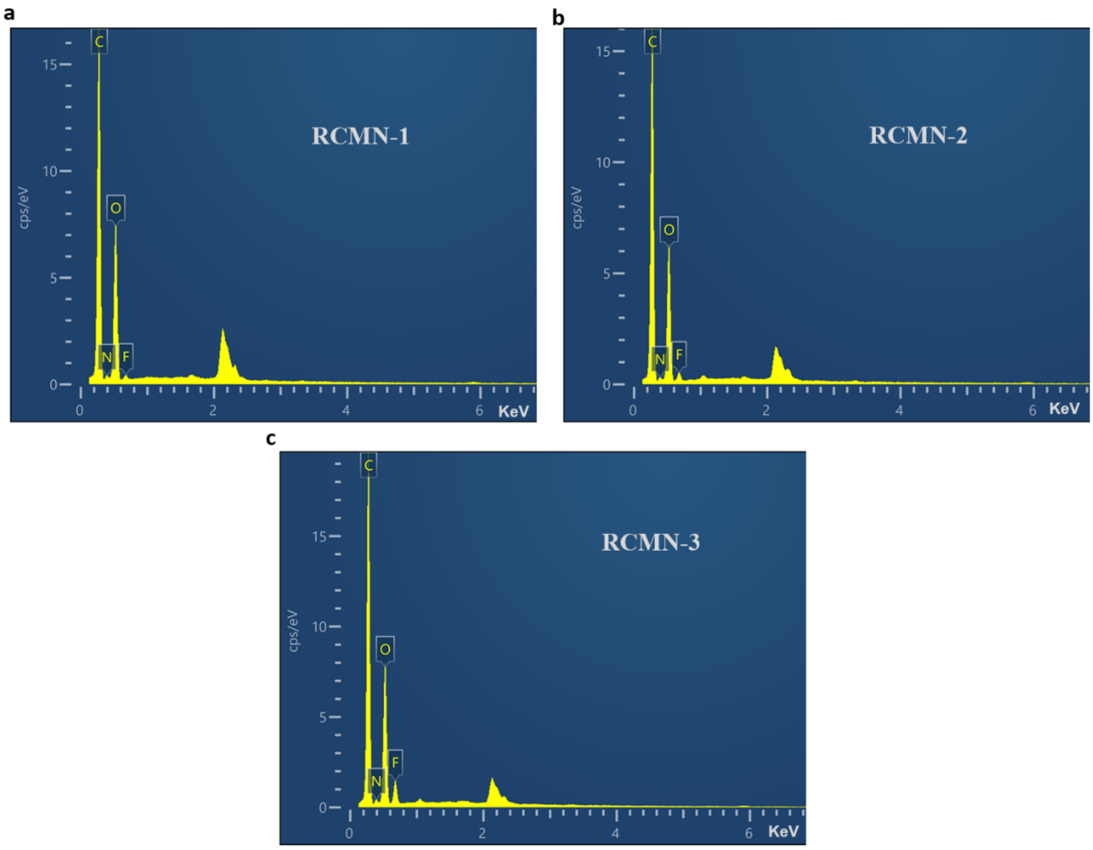

Figure S10. The Energy Dispersive Spectrometer (EDS) test of RCMN-1 (a), RCMN-2 (b) and RCMN-3 (c).

Table S3. The element composition (wt%) of RCMN-1, RCMN-2 and RCMN-3.

Sample	C	N	O	F
RCMN-1	57.70	3.36	38.39	0.55
RCMN-2	57.68	3.26	36.62	2.44
RCMN-3	55.56	4.02	34.52	5.90

The detailed element composition of RCMNs was obtained by SEM elemental mapping. Specifically, the fluorine contents (mass percent, wt%) of RCMN-1, RCMN-2 and RCMN-3 were 0.55, 2.44 and 5.90, respectively. The fluorine concentration could be expressed by the following equation:

$$[F] = \frac{\omega \cdot c_{RCMN} \cdot V}{M \cdot V} = \frac{\omega \cdot c_{RCMN}}{M} \quad \text{------ Eq. (2)}$$

where [F] (mol L^{-1}) is the fluorine concentration, ω (wt%) is the mass percent of fluorine in RCMN, c_{RCMN} (g L^{-1}) is the mass concentration of the corresponding nanoprobe (RCMN-1, RCMN-2 or RCMN-3) and M is the relative atomic mass of fluorine element (19 g mol^{-1}).

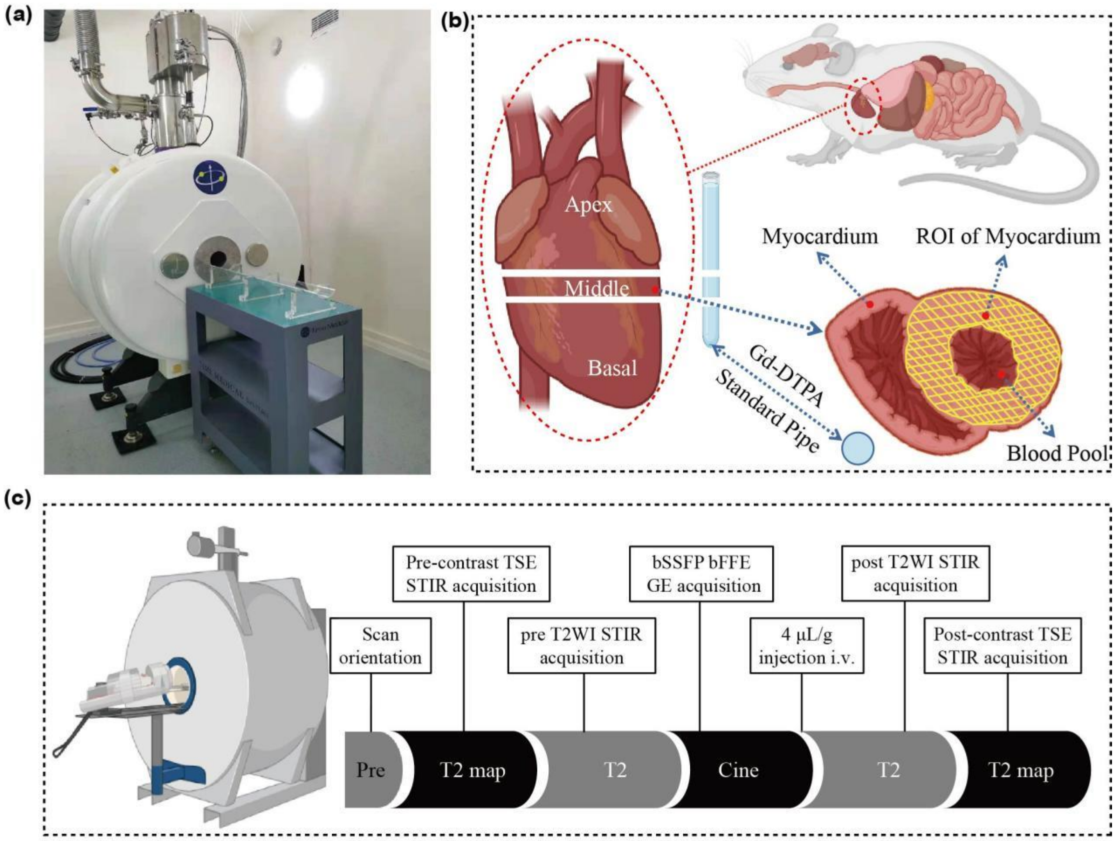

Figure S11. Schematic illustration of RCMNs for the early diagnosis of DCM via 7.0T MRI. (a) Image of the 7.0T MRI scanner. (b) Selection principles of myocardial region of interest (ROI). (c) Detailed scanning protocol for cardiac MRI. TSE, STIR, bSSFP, bFFE, GE, and T2WI are the name of the echoes used for the corresponding MRI sequence. i.v.: intravenous injection.

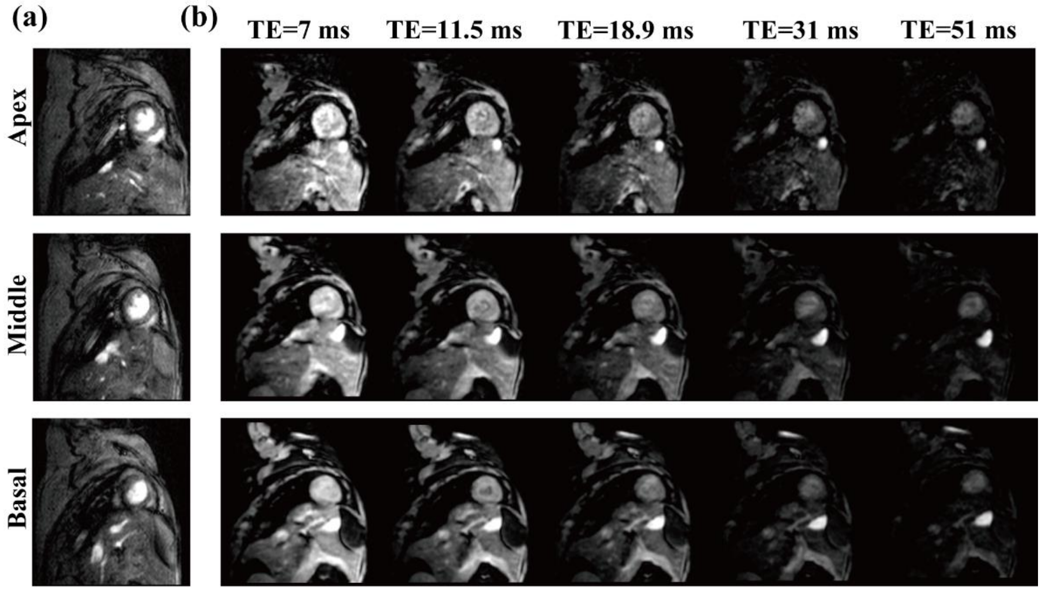

Figure S12. (a) Anatomic images for the localization of T_2 mapping and T2 black blood (3 slices). (b) Raw T_2 mapping images for a series of TEs (3 slices). The "Apex", "Middle" and "Basal" represent the apical, middle and basal slices of the heart, respectively.

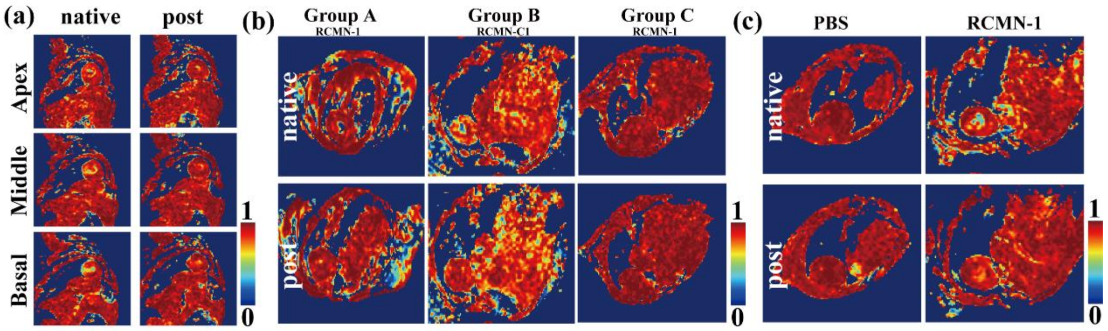

Figure S13. R-Squared map from T_2 fitting calculations of Figure 4b (a), Figure 4g (b) and Figure 5c (c).

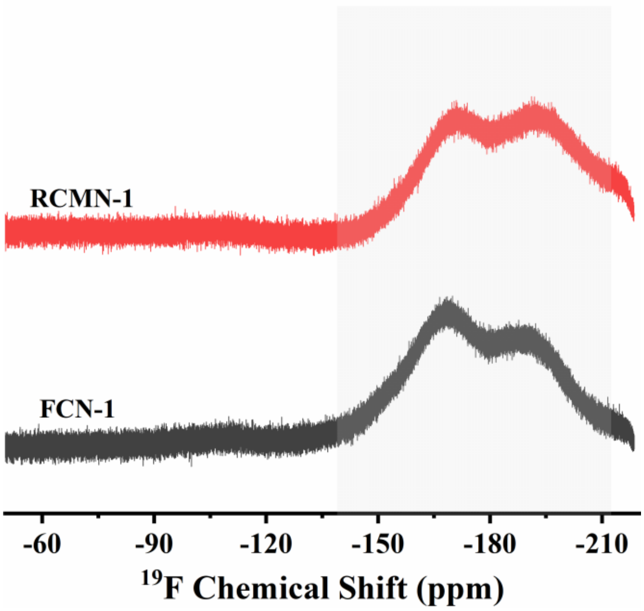

Figure S14. ^{19}F NMR spectrum of FCN-1 and RCMN-1.

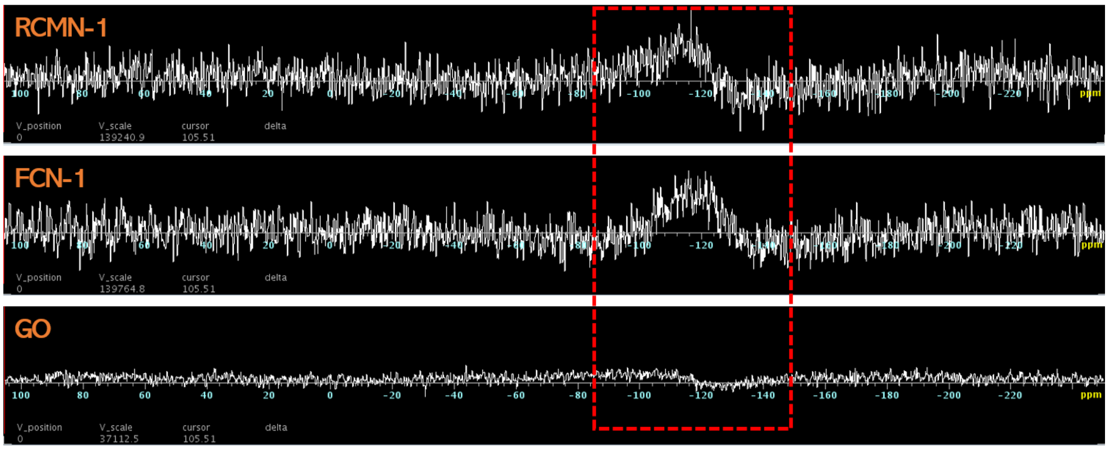

Figure S15. ^{19}F pre-scan spectrum using a 7.0 T MRI instrument.

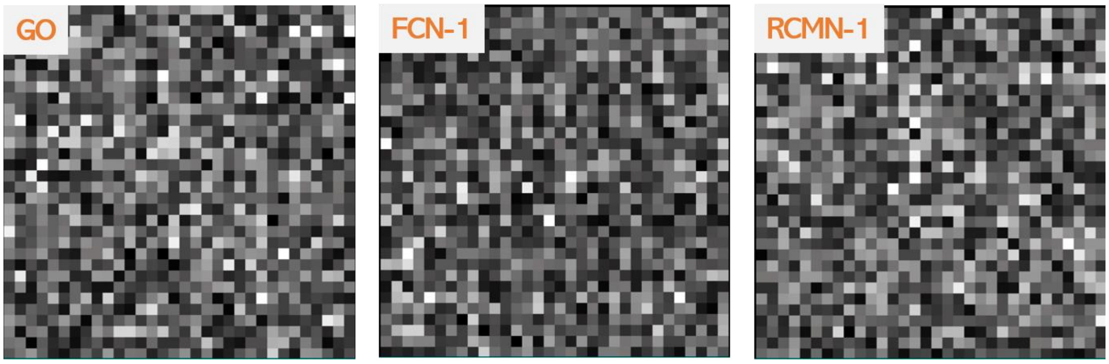

Figure S16. ^{19}F MRI images of GO, FCN-1 and RCMN-1 on a 7.0T MRI scanner.

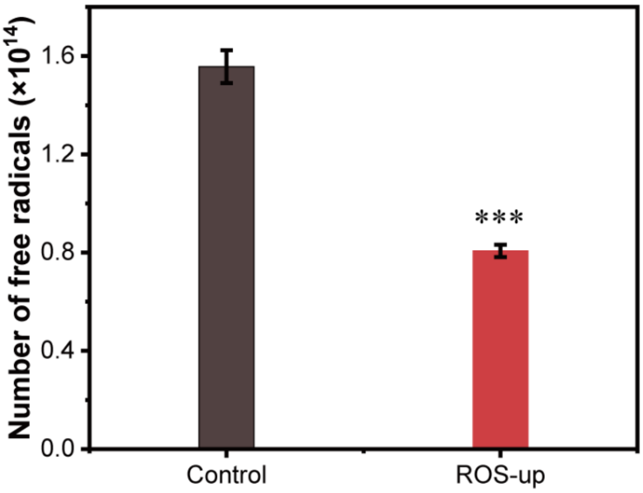

Figure S17. EPR scanning to quantify the number of free radicals. Data represents mean values ± SD, n = 3. Statistical differences were determined by unpaired Student's t-test. ***p < 0.001.

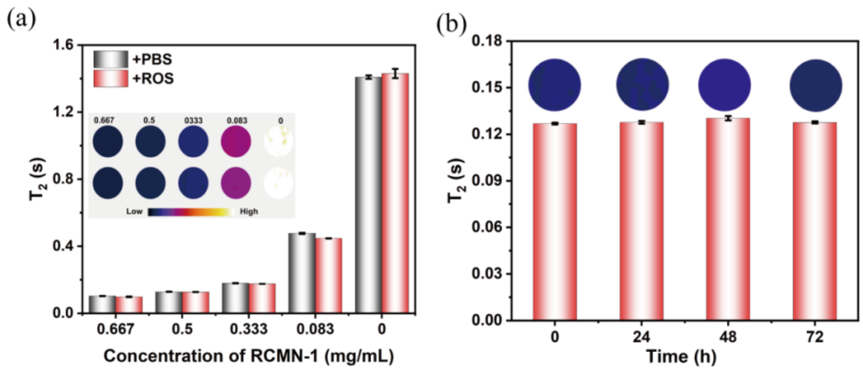

Figure S18. a) The T_2 relaxation time of RCMN-1 with different concentrations after adding ROS or PBS. b) T_2 MRI signal changes of RCMN-1 after addition of ROS (concentration of RCMN-1: 0.667 mg/mL).

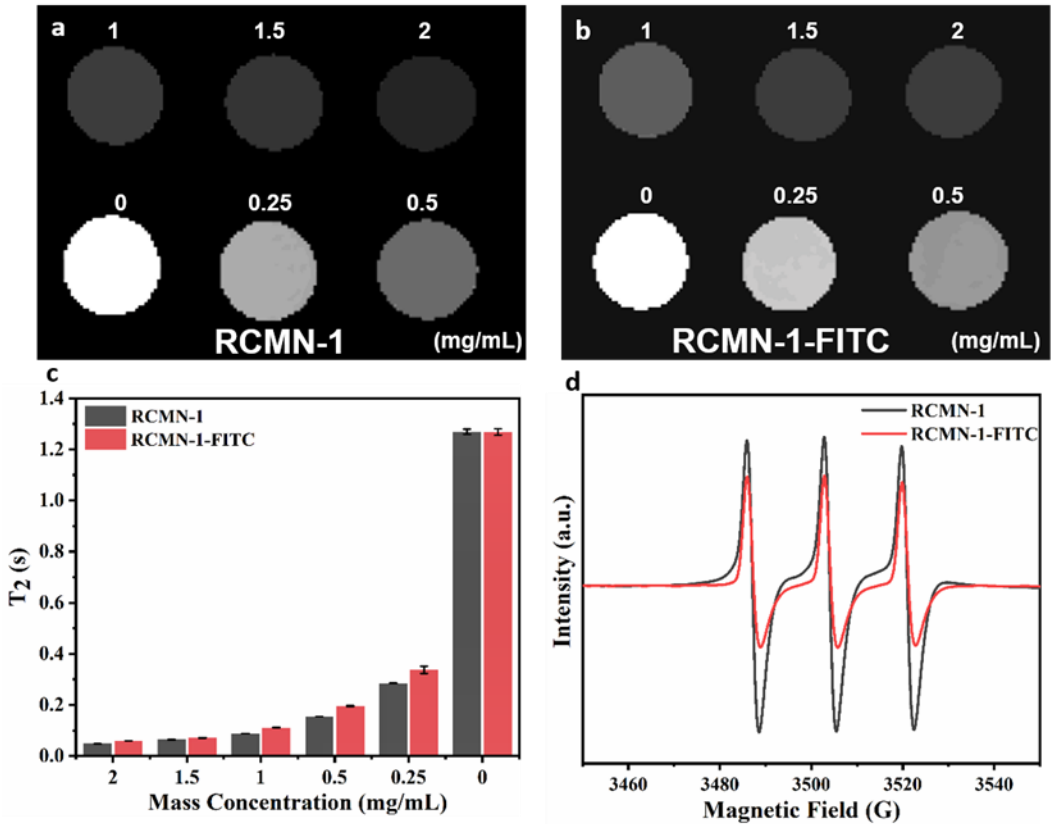

Figure S19. the changes of T2-weighted images of RCMN-1 (a) and RCMN-1-FITC (b) with their concentration. (c) The T2 relaxation time of RCMN-1and RCMN-1-FITC with different concentrations. (d) the changes of EPR signal intensity of RCMN-1 before and after introducing FITC.

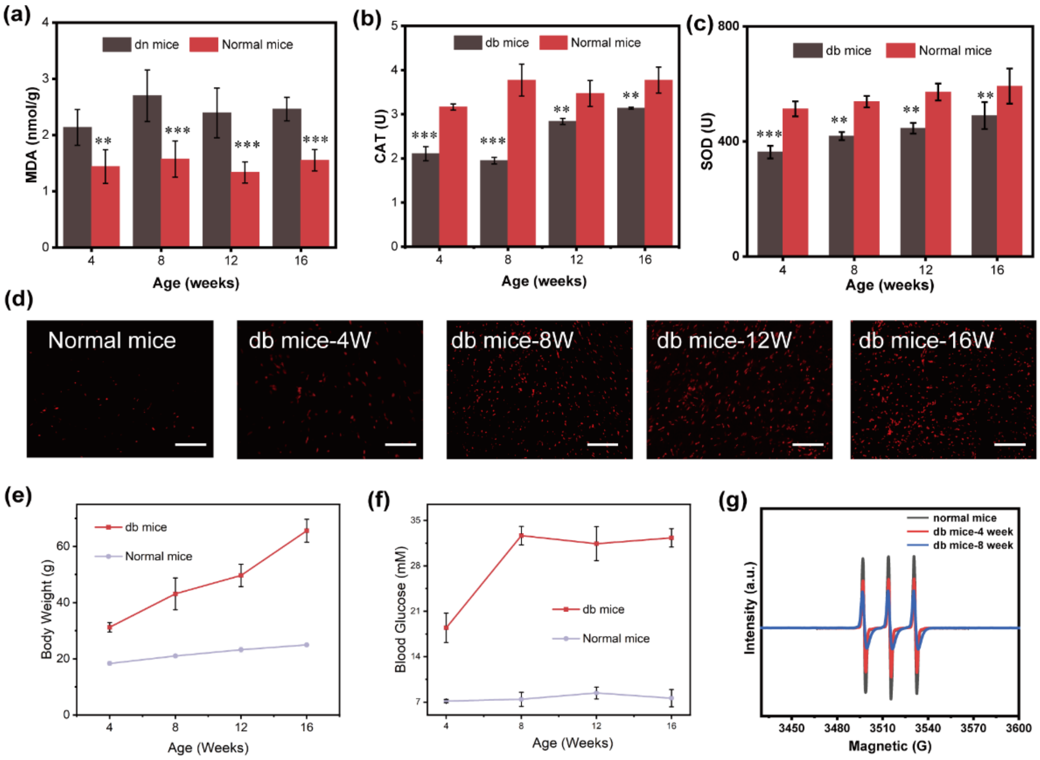

Figure S20. (a-c) Changes of oxidative stress indicators [lipid peroxidation products: malondialdehyde, MDA (a) and antioxidant enzymes: catalase, CAT (b); superoxide dismutase, SOD (c)]. d) ROS staining of mouse heart sections, scale bar: 50 μm. Body weight changes (e) and blood glucose changes (f) in normal mice and db mice. (g) EPR scanning of cardiac tissue in db mice and normal mice. Data in (a), (b), and (c) represent mean values ± SD, n = 10. Statistical differences were determined by unpaired Student's t-test. NS means no significant difference. $^{**}p < 0.01$, $^{***}p <$

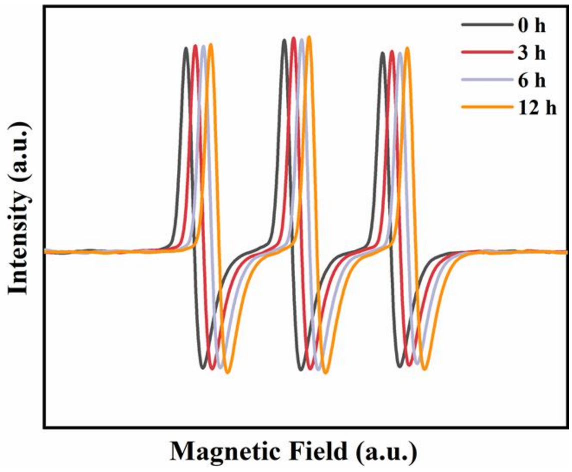

0.001.

Figure S21. EPR signal of RCMN-1 at different time points after mixing with serum.

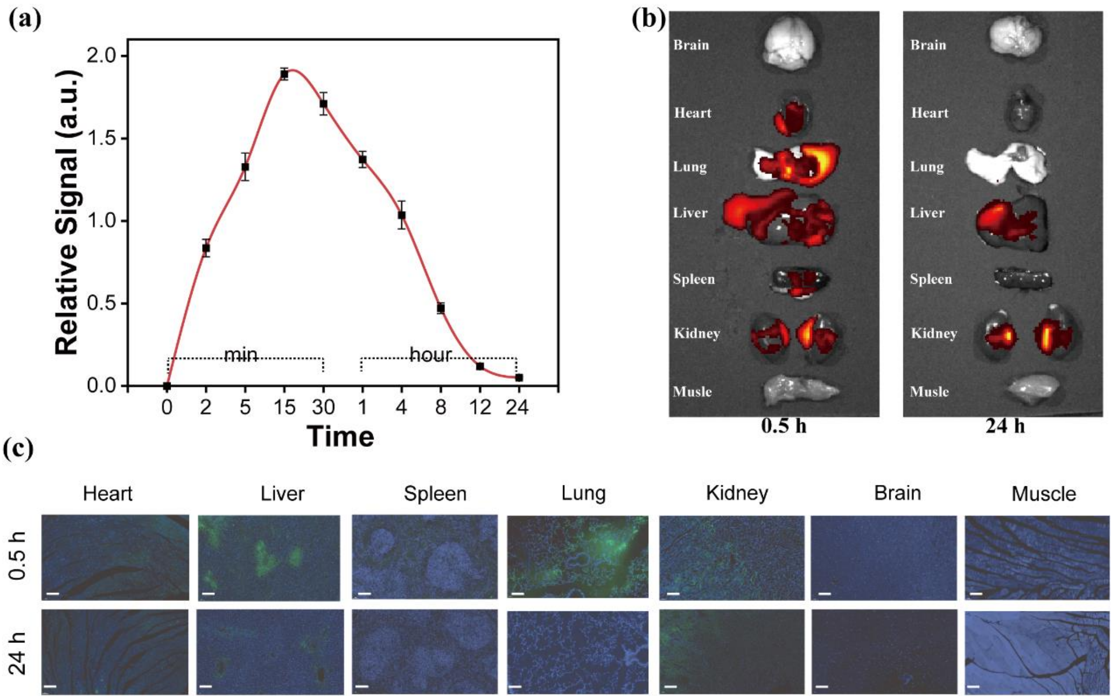

Figure S22. (a) The plasma drug concentration measurement. (b) Fluorescence imaging studies of mice organs at 0.5/24 h post injection of RCMN-1. (c) Confocal images of organ tissues sections at 0.5h/24h after tail vein injection of

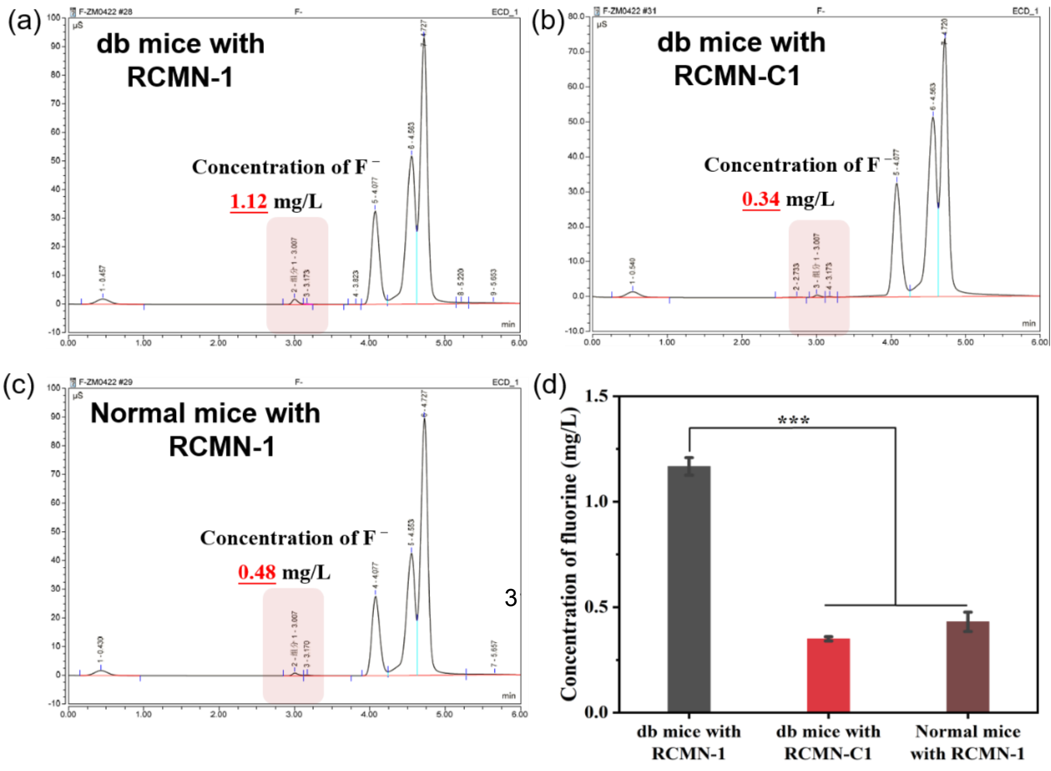

RCMN-1-FITC.

Figure S23. Ion chromatography of absorption solution of mouse-heart combustion products at 0.5 h post injection of RCMN-1/RCMN-C1. The fluorine contents of the hearts of db mice injected with RCMN-1 (a), db mice injected with RCMN-C1(b) and normal mice injected with RCMN-1 (c). (d) Statistical difference of the fluorine contents of the mouse-heart combustion products at 0.5 h post injection of RCMN-1/RCMN-C1. Data represent mean values ± standard deviations (SD), n = 3. Statistical differences were determined by unpaired Student's t-test. ***p < 0.001.

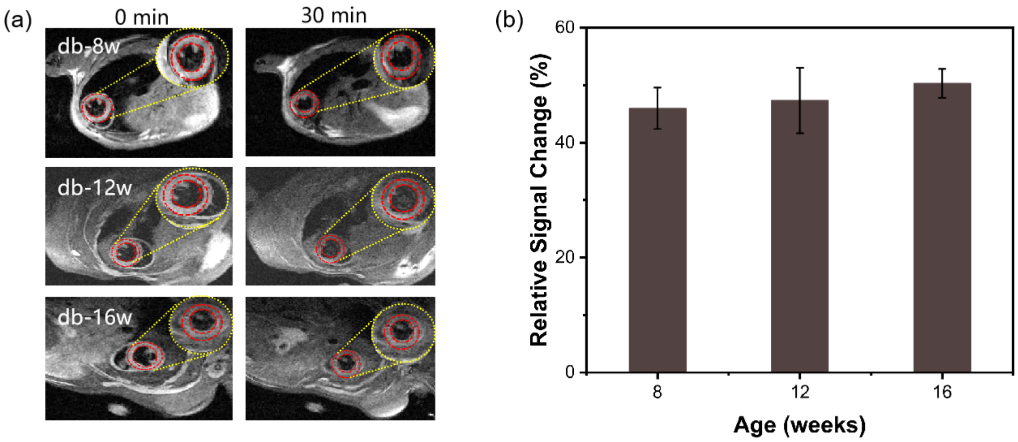

Figure S24. Heart T_2 black blood images (a) and signal values (b) of db mice injected with RCMN-1 at different ages.

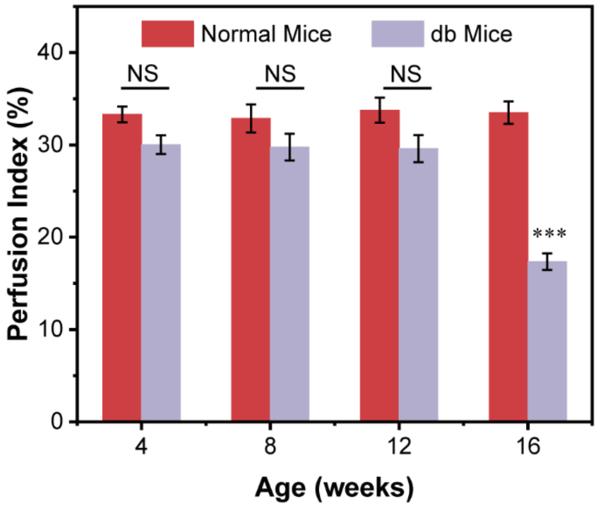

Figure S25. Comparison of the maximum slope of cardiac perfusion under stress between db mice and normal mice at different ages, Data represents mean values ± SD, n = 12. Statistical differences were determined by unpaired Student's t-test. NS means no significant difference. ***$p < 0.001$.

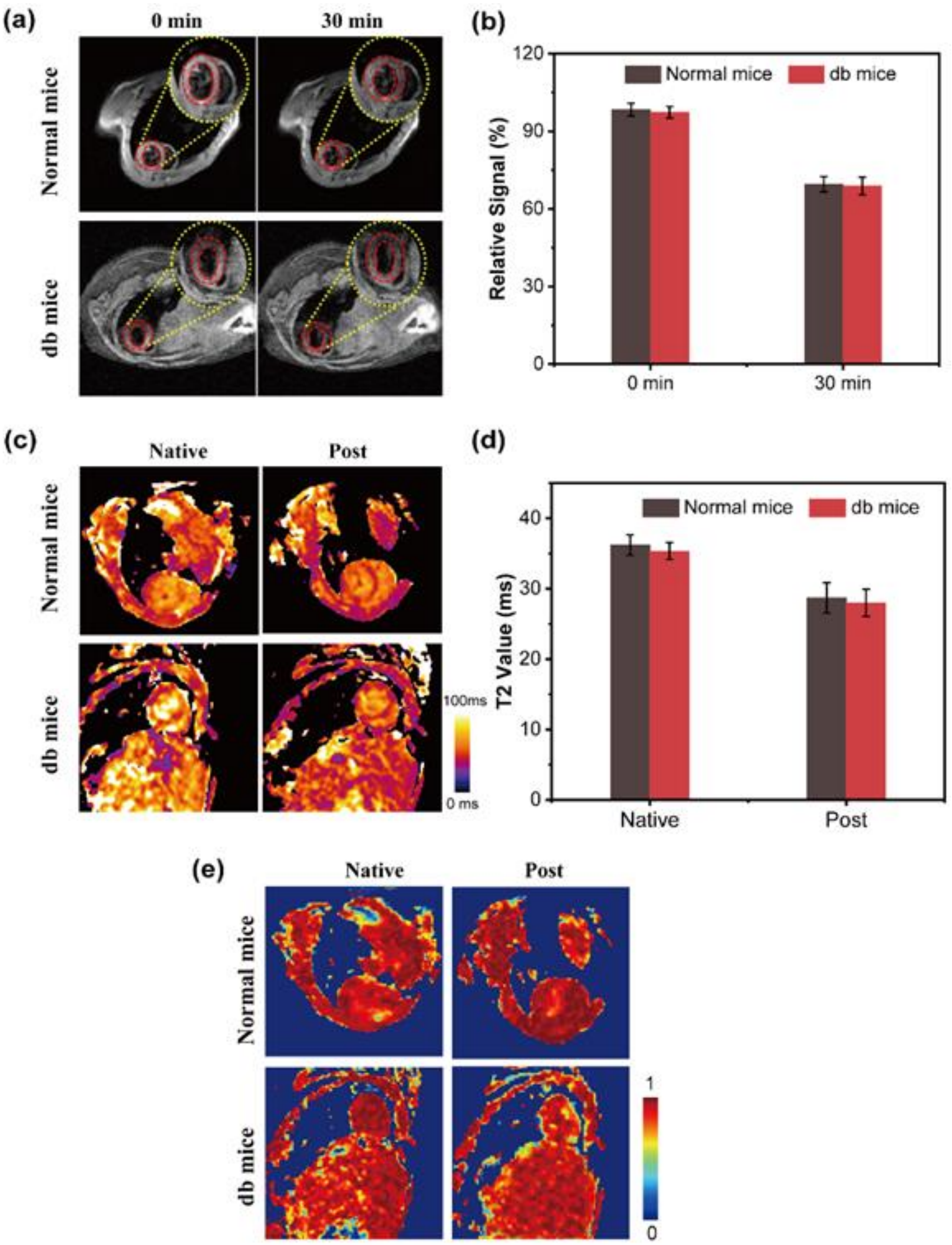

Figure S26. Cardiac MRI black blood images (a) and quantitative analysis (b), T2 maps (c) and T2 values (d) before and after probe injection. (e) R-Squared map from T2 fitting calculations.

Table S4. Left ventricular ejection fraction (LVEF) of normal mice and db mice at different ages.

	4W	8W	12W	16W
normal mice	71.35±5.21	71.03±3.48	72.65±6.21	72.19±6.38
db mice	73.25±4.18	72.36±4.01	70.28±5.14	70.25±3.81

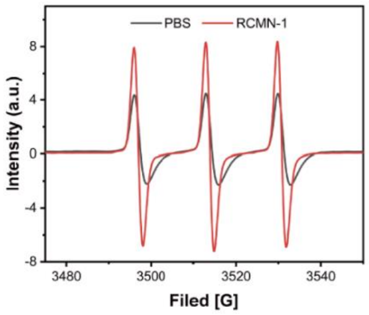

Figure S27. EPR signal of myocardium before and after treatment with different reagents (PBS or RCMN-1).

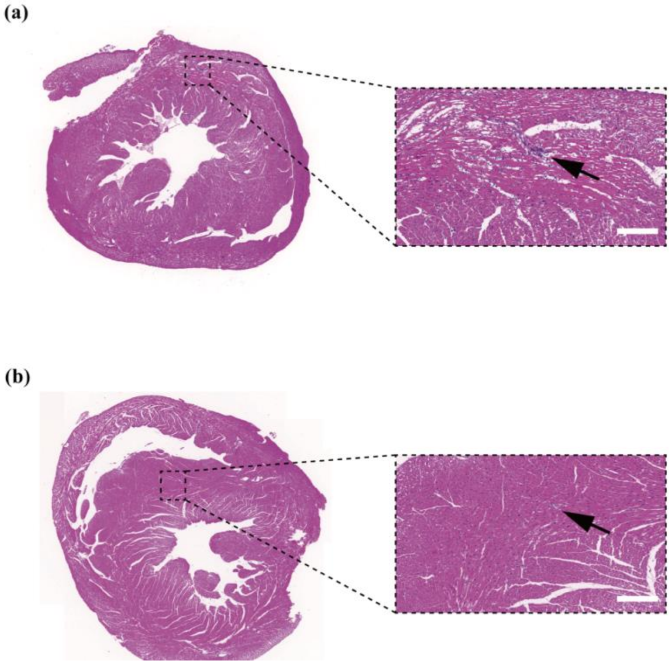

Figure S28. H&E staining of heart slices after administration of PBS (a) and RCMN-1 (b), scale bar: 50 μm.

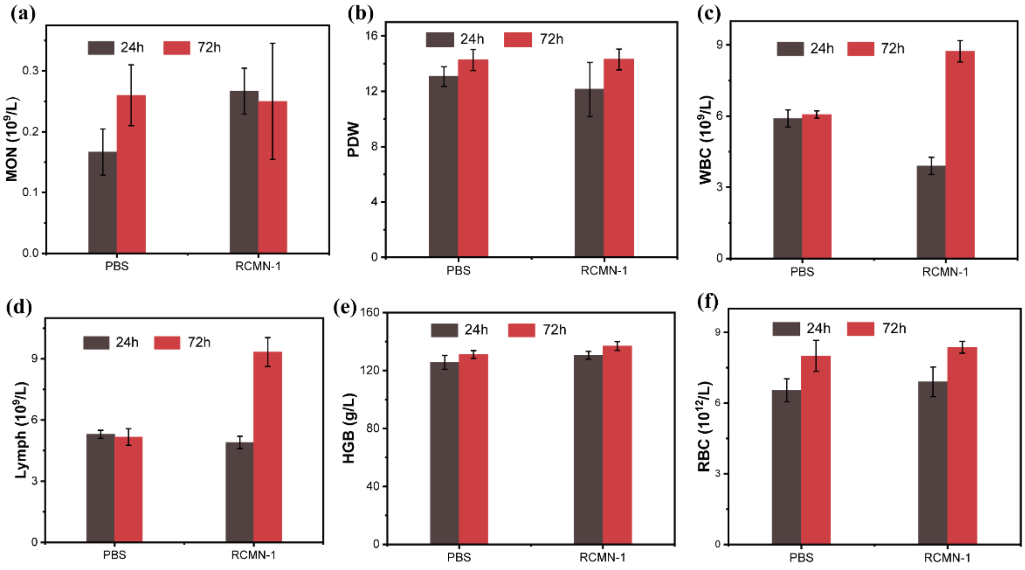

Figure S29. The whole blood analysis of the blood from the mice treated with RCMN-1 or PBS for 24 and 72 h.

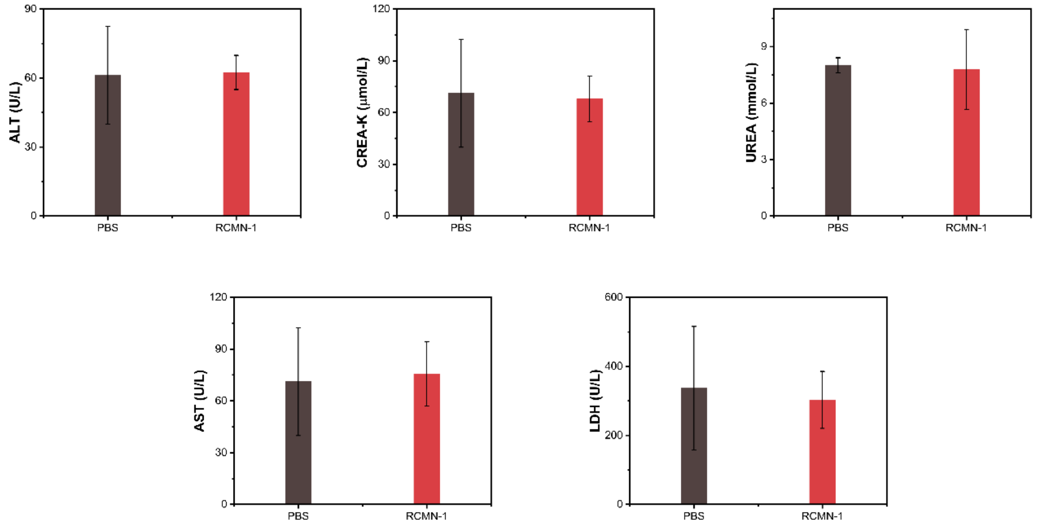

Figure S30. The liver, kidney and myocardium function analysis after intravenous injection of PBS or RCMN-1 nanoprobes.

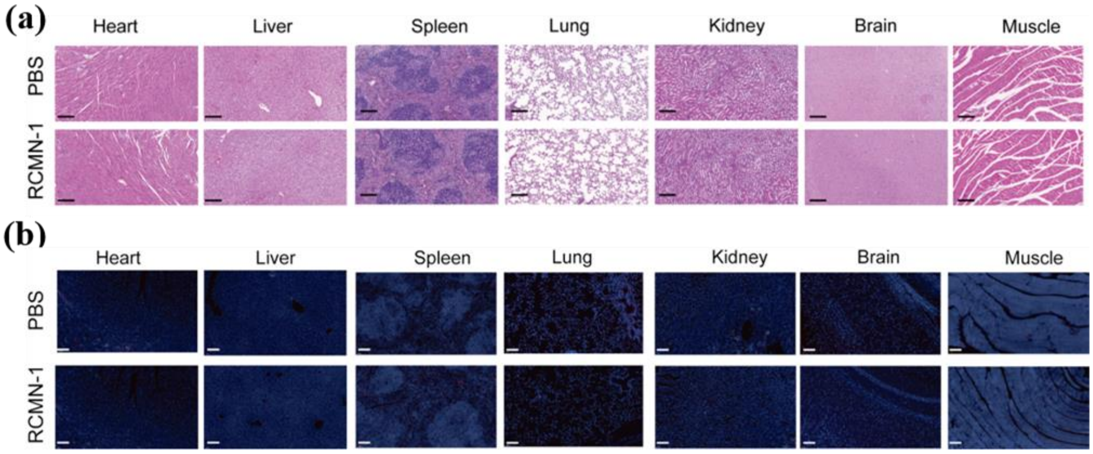

Figure S31. H&E staining (a) and TUNEL staining (b) of different organ tissues in mice, scale bar: 50 μm.

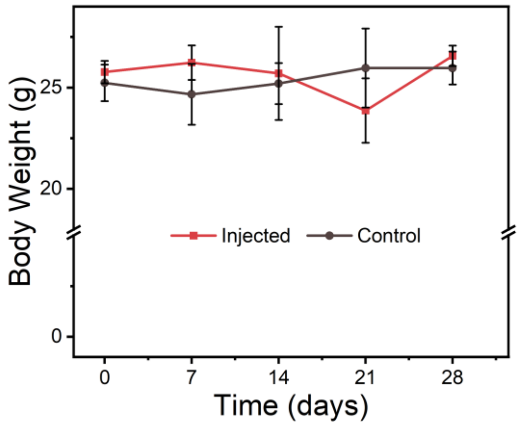

Figure S32. Changes of mice body weight after injection of PBS or RCMN-1.

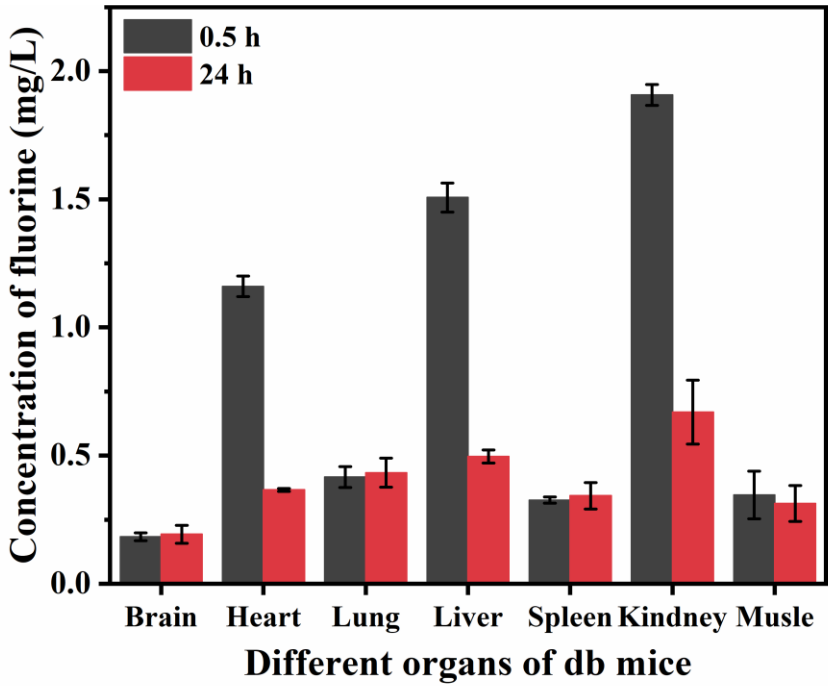

Figure S33. Fluorine contents (mg/L) of db mice (n=3) organs at 0.5/24 h post-injection of RCMN-1.
